# Dose-dependent effects of gamma radiation on the early zebrafish development and gene expression

**DOI:** 10.1371/journal.pone.0179259

**Published:** 2017-06-19

**Authors:** Selma Hurem, Leonardo Martín Martín, Dag Anders Brede, Eystein Skjerve, Rasoul Nourizadeh-Lillabadi, Ole Christian Lind, Terje Christensen, Vidar Berg, Hans-Christian Teien, Brit Salbu, Deborah Helen Oughton, Peter Aleström, Jan Ludvig Lyche

**Affiliations:** 1 Norwegian University of Life Sciences (NMBU), CERAD CoE, Faculty of Veterinary Medicine and Biosciences, Oslo, Norway; 2 University of Camagüey Ignacio Agramonte y Loynaz (UC), Faculty of Agropecuary Sciences, Camagüey, Cuba; 3 Norwegian University of Life Sciences (NMBU), CERAD CoE, Faculty of Environmental Sciences and Natural Resource Management, 1433 Ås, Norway; 4 Norwegian Radiation Protection Authority (NRPA), CERAD CoE, Østerås, Norway; Northwestern University Feinberg School of Medicine, UNITED STATES

## Abstract

Ionizing radiation from natural sources or anthropogenic activity has the potential to cause oxidative stress or genetic damage in living organisms, through the ionization and excitation of molecules and the subsequent production of free radicals and reactive oxygen species (ROS). The present work focuses on radiation-induced biological effects using the zebrafish (*Danio rerio*) vertebrate model. Changes in developmental traits and gene expression in zebrafish were assessed after continuous external gamma irradiation (0.4, 3.9, 15 and 38 mGy/h) with corresponding controls, starting at 2.5 hours post fertilization (hpf) and lasting through embryogenesis and the early larval stage. The lowest dose rate corresponded to recommended benchmarks at which adverse effects are not expected to occur in aquatic ecosystems (2–10 mGy/day). The survival observed at 96 hours post fertilization (hpf) in the 38 mGy/h group was significantly lower, while other groups showed no significant difference compared to controls. The total hatching was significantly lower from controls in the 15 mGy/h group and a delay in hatching onset in the 0.4 mGy/h group was observed. The deformity frequency was significantly increased by prolonged exposure duration at dose rates ≥ 0.4 mGy/h. Molecular responses analyzed by RNA-seq at gastrulation (5.5 hpf transcriptome) indicate that the radiation induced adverse effects occurred during the earliest stages of development. A dose-response relationship was found in the numbers of differentially regulated genes in exposure groups compared to controls at a total dose as low as 1.62 mGy. Ingenuity Pathway Analysis identified retinoic acid receptor activation, apoptosis, and glutathione mediated detoxification signaling as the most affected pathways in the lower dose rate (0.54 mGy/h), while *eif2* and *mTOR*, i.e., involved in the modulation of angiogenesis, were most affected in higher dose rates (5.4 and 10.9 mGy/h). By comparing gene expression data, *myc* was found to be the most significant upstream regulator, followed by *tp53*, *TNF*, *hnf4a*, *TGFb1* and *cebpa*, while *crabp2b* and *vegfab* were identified as most frequent downstream target genes. These genes are associated with various developmental processes. The present findings show that continuous gamma irradiation (≥ 0.54 mGy/h) during early gastrula causes gene expression changes that are linked to developmental defects in zebrafish embryos.

## Introduction

Living organisms are continuously exposed to ionizing radiation from naturally occurring radionuclides (e.g., radon daughters), cosmic radiation and from various anthropogenic activities (weapon testing, nuclear fuel reprocessing, nuclear accidents). Ionizing radiation interacts with matter by excitation and ionization of molecules, thereby producing free radicals and subsequently reactive oxygen species (ROS) and reactive nitrogen species (RNS) which can attack cell membranes or break chemical bonds in biological molecules, leading to oxidative stress or DNA damage [1]. Proliferating cells are specifically sensitive to radiation [2].

It is established that humans and animals are most vulnerable to ionizing radiation during early life stages such as gametogenesis, embryogenesis and organogenesis [3,4], due to the high rate of cell division, proliferation and differentiation. Ionizing radiation can affect all organs and biological systems, and can induce non-cancer effects as well as cancer [5]. Experimental studies have documented that exposure to ionizing radiation during critical periods of development may alter (reprogram) the differentiation signals leading to permanent toxic effects which can manifest later in life [5,6]. Permanent (irreversible) “developmental programming” is, among other mechanisms, attributed to epigenetic modification of gene transcription [7,8]. For aquatic organisms exposed to ionizing radiation, dose rates lower than 0.42 mGy/h (corresponding to 10 mGy/d) have been proposed as benchmark levels that are not likely to produce adverse effects at the population level [9]. Recently, a much lower predicted no effect dose rate (PNEDR) of 0.01 mGy/h (0.24 mGy/d) has been proposed as a risk assessment screening value below which one could be confident that exposures would not lead to adverse effects [10]. Protection criteria is based mostly on data from acute exposure experiments of adult organisms (IAEA), and the information on effects of ionizing radiation during sensitive life stages such as the embryonic and early larval development is scarce.

The zebrafish model offers many practical benefits, which may contribute to a better understanding of biological effects of radiation in both humans and non-human biota. Age-synchronized and optically transparent zebrafish embryos enable the visualization of major organ system development within all stages of the embryonic and early larval period. The available genomic resources in zebrafish, including a fully sequenced genome, have been proven valuable for providing various biological insights, including into human diseases [11]. Transcriptome analysis allows registration of changes in gene expression related to various biological processes and can be used to reveal potential mechanisms of radiotoxicity. The genome of the zebrafish is roughly half the size of the human genome and in comparison to it, shares about 70% of human gene orthologs and 82% of cancer gene orthologs [12,13].

This study aimed to assess biological effects such as survival, hatching and the occurrence of deformities in zebrafish exposed to gamma radiation (dose rates 0.4, 3.9, 15, and 38 mGy/h) and controls during embryogenesis and larval development (2.5 to 96 hpf). In order to elucidate the changes in gene expression with accompanying functional network analyses, RNA sequencing of total RNA extracted from irradiated (0.54, 5.4 and 10.9 mGy/h) pooled embryo samples and controls was performed. The embryos were exposed during 2.5–5.5 hpf, corresponding to the blastula period until the onset of the gastrula stage of development (256-cell stage until approximately 50% epiboly) [14]. This timeframe also includes the zygotic genome activation (ZGA) and onset of cell specification takes place [15–17]. The early molecular events initiated by a very low total dose of gamma radiation at 5.5 hpf and analyzed by transcriptomics were shown to be consistent with the observed developmental adversity in later stages.

## Materials and methods

### Ethics statement

The research was carried out according to the Norwegian Animal Protection Act (implemented EU Directive 2010/63/EU) and approved on December 12, 2013 by IACUC at Norwegian School of Veterinary Science (since 2014 Norwegian University of Life Sciences, Faculty of Veterinary Medicine and Biosciences, Oslo, Norway), under approval number FOTS ID 5793.

### Zebrafish maintenance and embryo handling

Embryos from the AB wild type strain were obtained from the NMBU zebrafish facility (Norwegian Zebrafish Platform) and maintained according to standard operating procedures. Zebrafish were kept at 28 ± 1°C on a 14–10 hour light-dark cycle at a density of 5–10 fish/L. The system water (SW) was prepared from particle and active charcoal filtered reverse osmosis (RO) deionized tap water, kept sterile by UV irradiation. To generate a conductivity of 500 μS/cm, general hardness (GH) of 4–5 and pH 7.5 (adjusted with 1M HCl), 155 mg synthetic sea salt (Instant Ocean, Blacksburg, USA), 53 mg sodium carbonate and 15 mg calcium chloride (Sigma-Aldrich) was added per liter RO water. Adult fish were fed with Gemma Micro 300 (Skretting, Stavanger, Norway) dry feed twice a day and live artemia (Scanbur, Copenhagen, Denmark) once a day. Health was monitored by daily inspection, sentinel fish were sent to ZIRC for pathology every six month and water sent for microbiology analysis (NMBU Vetbio, Oslo). Adult fish were allowed to mate for 30 min in standard 1 L breeding tanks (Aquatic Habitats, Apopka, FL). For gamma radiation experiments, embryos were collected immediately after breeding and individually placed in 2 first rows of replicate 96 well plates (Nunc™, Thermo-Fisher Scientific, Waltham, Massachusetts, USA) with 200 μl of egg water (temperate autoclaved system water). A second group of embryos was placed in 2.5 ml tubes (Thermo-Fisher Scientific, Waltham, Massachusetts, USA) (50 embryos/ tube) with 2 ml egg water.

### Embryo irradiation and dosimetry

After collection, embryos were transported to the FIGARO experimental irradiation facility at NMBU, Ås, Norway (^60^Co source, activity ~ 420 GBq). For both the toxicity endpoints and transcriptomic analyses, external gamma irradiation of zebrafish embryos commenced at 2.5 hpf with total doses to water ranging from 1.62 mGy– 3496 mGy during a 3 hour, 43.8 hour and 92 hour irradiation timespan (Table 1). Dose rates of 0.4, 3.9, 15 and 38 mGy/h were used for general toxicity analyses, and 0.54, 5.4 and 10.9 mGy/h for the transcriptomic analyses (Table 1). The experiments for both analyses were performed at separate time intervals. All exposures included corresponding controls. For the adverse effect observations and RNA-seq, 96-well plates and 2.5 ml tubes, respectively, were positioned at different distances from the gamma source corresponding to the dose rates to water (D_Water_) presented in Table 1.

**Table 1:** Exposure groups and dosimetry. Total doses from measured dose rates during different time periods of exposure. **(A)**: 43.8 hours; **(B)**: 92 hours and **(C)**: 3 hours.

**Developmental traits**	**Dose rate D**_**Water**_ (mGy/h)*		**0.4**	**3.9**	**15**	**38**
	**Total dose D**_**Water**_ **(mGy)**	**(A)**	17.5	171	657	1664
		**(B)**	36.8	359	1380	3496
**RNA-seq**	**Dose rate D**_**Water**_ (mGy/h)*		**0.54**	**5.4**	**10.9**	
	**Total dose D**_**Water**_ **(mGy)**	**(C)**	1.62	16.2	32.7	

*Uncertainty (K = 2) for dose rate estimates is ~10% (Bjerke and Hetland, 2014).

Field dosimetry (air kerma rates measured with an ionization chamber) was traceable to the Norwegian Secondary Standard Dosimetry Laboratory (Norwegian Radiation Protection Authority, NRPA, Oslo, Norway) [18]. Average dose rates to water in the first and second rows of microplate wells were estimated according to established technical guidelines [19] and used as a proxy for dose rates to the fish embryos. Controls were placed in the same room, outside the beam cone and shielded by lead reducing the external (background) dose rate to ≤ 0.35 μGy/h (Thermo Eberline FHT6020). The irradiation room was thermostatically heated (28 ± 2°C), and had a 14–10 hours light-dark cycle (250–320 lx). To minimize variation in temperature, 2 control groups were used for the transcriptomic analyses.

### Sampling procedure and experimental analysis of general toxicity endpoints

At approximately 48 hpf, half of the 96-well plates were removed from exposure (Table 1, Group “A”), while the remaining embryos were irradiated until 96 hpf (Table 1, Group “B”), n ≥ 145/ group. To determine the general toxicity in terms of adverse effects on survival and hatching, the embryos and larva were manually observed in a stereo microscope (3.5–45x) at 48 and 96 hpf in group “A”, and at 96 hpf in group in “B” (S1 Table). Additionally, the occurrence of deformities was observed at 96 hpf in both “A” and “B”. Analysis of endpoints was performed according to observations guidelines [20]. After observations, the larva used in this study was euthanized (prior to independent feeding at 120 hpf) using Tricaine (MS-222) (Sigma-Aldrich) overdose followed by rapid freezing (-70°C). For RNA extraction, embryos were sampled at 5.5 hpf (Table 1, Group “C”) in 2.5 ml tubes (n = 50/ sample).

### Transcriptome analysis at 5.5 hours post fertilization

RNA sequencing was conducted to compare gene expression profiles between the controls and the 0.54, 5.4 and 10.9 mGy/h exposed embryos. Total RNA was isolated from embryos exposed between 2.5 hours and 5.5 hpf with TRIzol Reagent (Invitrogen) and purified with RNeasy Mini Kit (Qiagen) according to manufactures’ instructions. Briefly, 1 ml TRIzol was added to each sample consisting of 50 embryos and homogenized using Magnalyser Beads (Roche Diagnostics). Isolated RNA was DNase I (Qiagen) treated for 20 min at 25°C before further purification. Each sample was eluted in 50 μl RNase-free water and stored at − 80°C until required. RNA purity and yield (A260/A280 > 1.8, A260/A230 > 2, yield > 200 ng/μl) was determined using NanoDrop-1000 Spectrophotometer (NanoDrop Technologies, Wilmington, DE) and quality (RIN > 8.5) was assessed with Agilent 2100 Bioanalyzer (Agilent Technologies, Palo Alto, CA) using RNA Nano LabChip Kit (Agilent Technologies). None of the samples showed any signs of degradation or impurities. Photometric parameters and RNA integrity number (Bioanalyzer; Agilent technologies, USA) determined the quality of RNA sequenced samples. The RNA was sequenced (Illumina HiSeq 2000) at BGI Tech Solutions Co., Ltd., Hong Kong. Three single-end libraries (biological replicates), in the 5.4 and 10.9 mGy/h groups and a duplicate per 0.54 exposure group were sequenced. The bioinformatics analysis pipeline of the RNA sequencing data is presented in S1 Fig. Quality assessment of raw reads (49 nt long) and adapter trimming was performed using Trim Galore! v0.3.7, a wrapper tool around Cutadapt and FastQC to consistently apply quality and adapter trimming to FastQ files [21,22]. Only reads with Phred score > 20 were kept. Afterwards, using TopHat v2.0.9 [23] with bowtie1, reads were mapped to the ZF genome (version Zv9, release 76) downloaded from Ensembl (http://www.ensembl.org/Danio_rerio/Info/Index). Options -g (maximum multihits number) was modified from its default value to 1, --no-coverage-search was allowed, --library type was set to “fr-unstranded” and -p (number of threads) was restricted to 4. As for bowtie1 options, -q (fastq files), -v (report end to end), -k 20 (report up to 20 good alignments), -m 20 (suppress all alignments if > 20), -S (to use SAM format) were used. BAM files were uploaded into Seqmonk [24] for visualization of aligned and mapped reads and read counting. Reads were counted as reads exactly overlapping with exons and the resulting count table was analyzed for gene expression under edgeR v3.4.2 Bioconductor [25]. The RNA-seq experiment was deposited in SRA database (https://www.ncbi.nlm.nih.gov/) and is publically available under accession SRP096352.

### Quantitative real-time PCR (qPCR) analysis

To verify the RNA-sequencing results, eight differently expressed genes were selected for qPCR analysis, based on their common differential expression in the exposure groups. The DNA Sequence information for each gene was retrieved from genebank (http://www.ncbi.nlm.nih.gov/Genbank). The Primer3Plus software (http://www.bioinformatics.nl/cgi-bin/primer3plus/primer3plus.cgi/) was used to design primers. These primers were analyzed for oligo duplex and primer dimers. Amplicons which are shorter than 130 bp and spanned over different exons were selected (S1 Table). The cDNA was prepared from 1 μg of same total RNA used for RNA sequencing analyses (n = 3). For cDNA synthesis, Superscript III reverse transcriptase (Invitrogen, USA) and random hexamer primers were used according to product specifications. The qPCR was performed on a LightCycler^®^ 96 Real-Time PCR system (Roche, Mannheim, Germany) using LightCycler^®^ 480 SYBR Green I Master (Roche). Each cDNA was analyzed in a duplicate and composed of 5 μL mastermix, 2 μL primer mix (5 μM of each forward and reverse), and 3 μL of each 10× diluted cDNA sample in a total volume of 10 μL. The cycling parameters were 10 min pre-incubation at 95°C, followed by 45 cycles of amplification at 95°C for 10 sec, 60°C for 10 sec and 72°C for 8 sec, followed by a melting curve from 60°C to 95°C. The qPCR assay was performed in three biological replicates. RefFinder analysis tool (http://fulxie.0fees.us/) [26] was used to find the best candidate reference genes. Analyzed reference genes were *hmbs* (hydroxymethylbilane synthase), *b-actin* (beta-actin) and *rps18* (ribosomal protein S18). For all exposure groups, *hmbs* was found to be the most stable housekeeping gene. The expression of each target gene transcript was normalized to *hmbs* and the fold change was calculated using the ΔΔCT method.

### Ingenuity pathway analysis

For predicted networks/pathways and biological function analyses of differently transcribed genes, IPA software (http://www.ingenuity.com, Ingenuity Systems Inc., Redwood City, CA) was used. The Core analysis and comparison sub analysis blocks were used to determine the interaction networks of up- and down-regulated genes, upstream regulators and biological states (diseases and bio functions) in each and across the three exposure doses. A right-tailed Fisher’s exact test was used to determine the probability that each biological function is due to chance alone and the association identified as statistically significant and non-random (p < 0.05). The results in gene regulation are given as negative logarithms of the p-value computed by numbers of genes participating in the process, number of genes from the reference dataset mapped to the network and the size of the entire network in the Ingenuity knowledge database. The upstream regulator analysis examines how many known targets of each transcription regulator are present in the dataset, comparing their expression to what is known from the literature. In the present study, ranking by overlap p-value (cutoff p-value ≤ 0.001) and filtering for genes, RNAs and proteins in order to predict the most relevant transcriptional regulators was used. For the predicted activation state of the transcription regulators, a z-score describing the quantity of activated (z-score > 0) or inhibited predictions (z-score < 0) was calculated. However, this prediction is not available for upstream regulators with less target genes in the datasets (i.e. in lower dose-rates), and could not be considered to determine the most likely relevant regulators where the value of the correction for the z-score was too high (bias > 0.25).

### Statistical analyses

After establishing the database for the general toxicity observations, tabulating and checking for errors in Excel^®^, data were transferred to Stata (MP/14 for Windows, StatCorp, College Station, TX). Confidence intervals were calculated using the proportion command for each of the outcomes survival, hatching and deformities at dose levels and the two exposure durations. Further logistic regression reported as odds ratios (OR) was used to estimate the effect of the treatments on hatching, survival and deformities and standard methods were used to check model fit. If significant, multiple comparisons were conducted using Tukey’s or Dunnett’s tests (Graphpad Prism 6, La Jolla, USA). Statistical significance was set to p < 0.05.

For analysis of gene expression, the dataset was TMM normalized first (trimmed mean of M-values, edgeR v3.4.2 Bioconductor, Robinson, McCarthy, and Smyth 2010), followed by data exploration using the statistical package R v3.0.2 [27]. Data was explored for descriptive statistics such as: minimum, maximum, 1^st^ quantile, 3^rd^ quantile, median, mean, standard deviation, also the similarity among samples was determined by correlation analysis and hclust (ward method) analysis to determine the distance between samples. The statistical analysis of differentially expressed genes (DEGs) was based on pairwise comparison between treatment and control RNA-seq samples (biological replicates) with a cut off set to ± 0.40 log2 fold change (1.3 FC). The FDR (false discovery rate) was set up to a significance of p ≤ 0.05. Venn diagram (Venny v2.1, Oliveros, (2007–2015)) was used to explore overlapping differential expressed genes among radiation treatments. For qPCR, obtained mean relative gene expression values (exposed vs. control) were compared to mean relative gene expression values for the same genes from RNA-seq and a Pearson’s correlation coefficient was calculated (p < 0.05) for all three exposure groups (Graphpad Prism 6, La Jolla, USA).

## Results

### General toxicity

**Fig 1:**
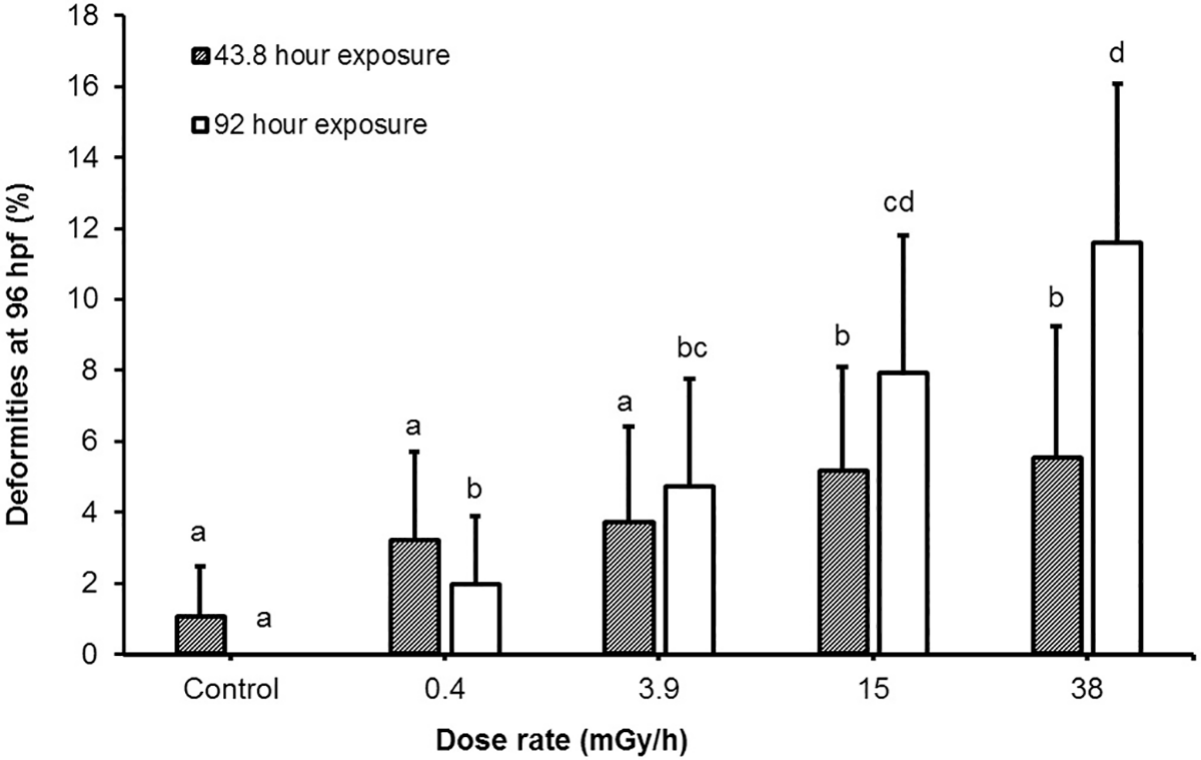
Deformities. Deformities observed at 96 hpf which occurred after a 43.8- and 92-hour exposure to the specified dose rates. The exposures had separate controls. Values presented as mean percentage ± 95% confidence interval (p < 0.05).

To determine the effects of gamma radiation on the embryonic and larval development, the survival, hatching rate and deformities were assessed at 48 and 96 hpf. Compared to controls, a decrease in survival was observed in all exposed groups, albeit only the 38 mGy/h group was statistically significant, both after a 43.8-hour and 92-hour exposure (Table 2, S2 Table). The timing of hatching was significantly affected by irradiation, as a premature onset of hatching in the 0.4 mGy/h group (p < 0.0001) and a delayed onset of hatching in the 38 mGy/h group (p = 0.0072), respectively, were observed (S2 Table). The total hatching was above 95% in all exposure groups, however, with significantly lower total hatching in fish exposed to 15 mGy/h compared to controls (Table 2, S2 Table). The deformity frequency at 96 hpf increased linearly in response to dose for both the 43.8- and 92-hour exposure (linear regression, R^2^ = 0.93 and R^2^ = 0.99, respectively) and was significantly higher than in controls (p < 0.05) in all exposure groups, except from the 43.8-hour exposure to 0.4 mGy/h and 3.9 mGy/h (Fig 1, Table 2). The lowest dose rate (0.4 mGy/h) caused significant increase in deformities (p = 0.049) only in the group exposed for 92 hours (Fig 1, Table 2). The most frequently observed deformities were retardation in development manifested as failed hatching and absence of pigmentation, irregularities in formation of the head and eyes, as well as a short tail or lack of a tail (S15 Fig). In summary, a significant dose dependent response was observed for deformities and mortality, whereas hatching showed a non-monotonic dose-response.

**Table 2:** Adverse effects. Adverse effects on total hatching, survival and deformities at 96 hpf, reported as Odds Ratios with 95% confidence intervals and related p-values compared to the base level (OR = 1). The OR describes the risk for occurrence of an adverse effect, given the two variables: dose rate and duration of exposure to the specified dose-rates. Significance denoted with (*).

Odds ratio (95% CI); p-values compared to base level				
Variables		Hatching	Survival	Deformities
**Dose rate (mGy/h)**	**Control**	1.00 (-)	1.00 (-)	1.00 (-)
	**0.4**	0.40 (0.08–2.10); 0.28	0.65 (0.41–1.04); 0.07	5.00 (1.09–23.0); 0.04*
	**3.9**	0.39 (0.08–2.03); 0.26	0.66 (0.42–1.06); 0.09	8.44 (1.93–37.0); 0.005*
	**15**	0.13 (0.03–0.59); 0.008*	0.75 (0.47–1.20); 0.23	13.43 (3.16–57.0); <0.001*
	**38**	0.26 (0.05–1.24); 0.09	0.46 (0.29–0.73); 0.001*	18.4 (4.37–77.6); <0.001*
**Duration of exposure (hours)**	**43.8**	1.00 (-)	1.00 (-)	1.00 (-)
	**92**	0.77 (0.45–1.33); 0.35	0.99 (0.78–1.27); 0.98	1.61 (1.09–2.37); 0.015*

### Gene expression analysis

**Fig 2:**
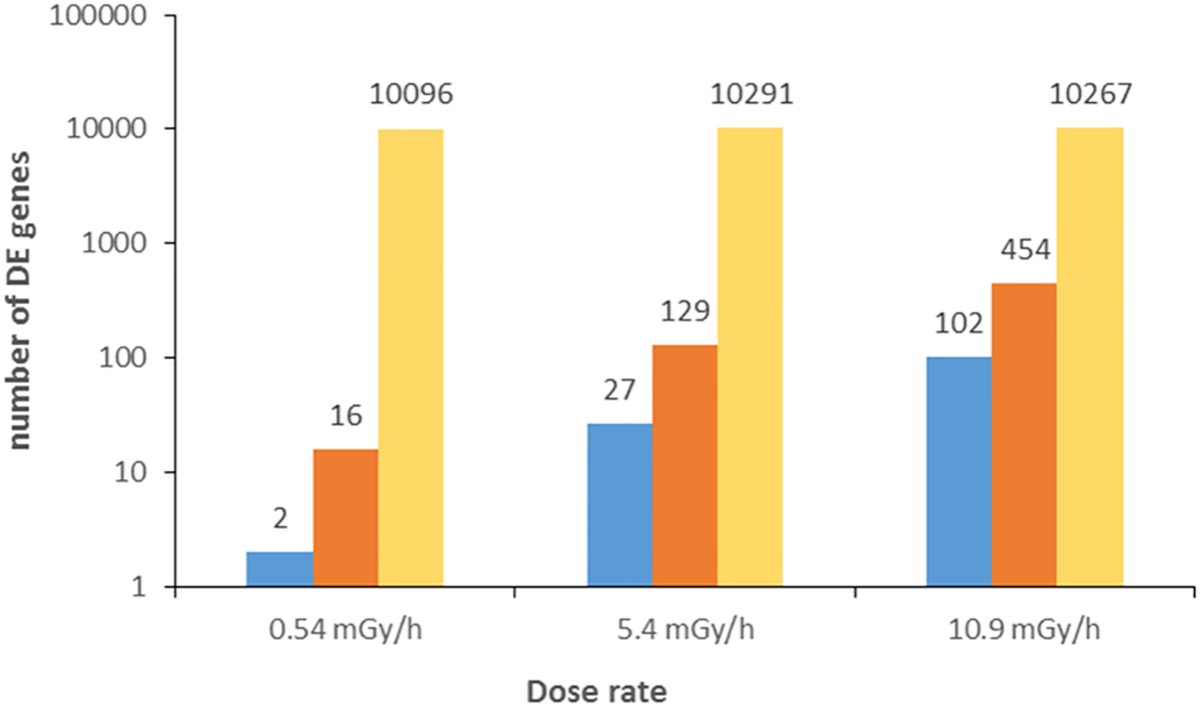
Expressed and differentially expressed genes in each exposure. Threshold set to FC ±1.3 FDR < 0.05; down-regulated genes (blue), up-regulated genes (red) and total number of expressed genes (yellow).

Transcriptional analysis was performed at the gastrula stage 5.5 hpf in order to identify potential changes to the transcriptional program induced by the gamma exposures. An average of 27 million reads (49 nt long) were obtained in both the treated and control groups. The mapping statistics showed a high grade of similarity among all samples (S2 Fig, S3 Table). The expression dataset analysis for replicability and distribution by means of multidimensional scaling plot (MDS) showed a clear difference between exposed and their respective controls (S3 Fig). A total number of ~10000 genes was expressed in all samples, while the number of differentially expressed genes (DEGs) showed a clear dose rate dependency (Fig 2 and S4 Fig and the full list of DEGs is available in S4 Table).

**Fig 3:**
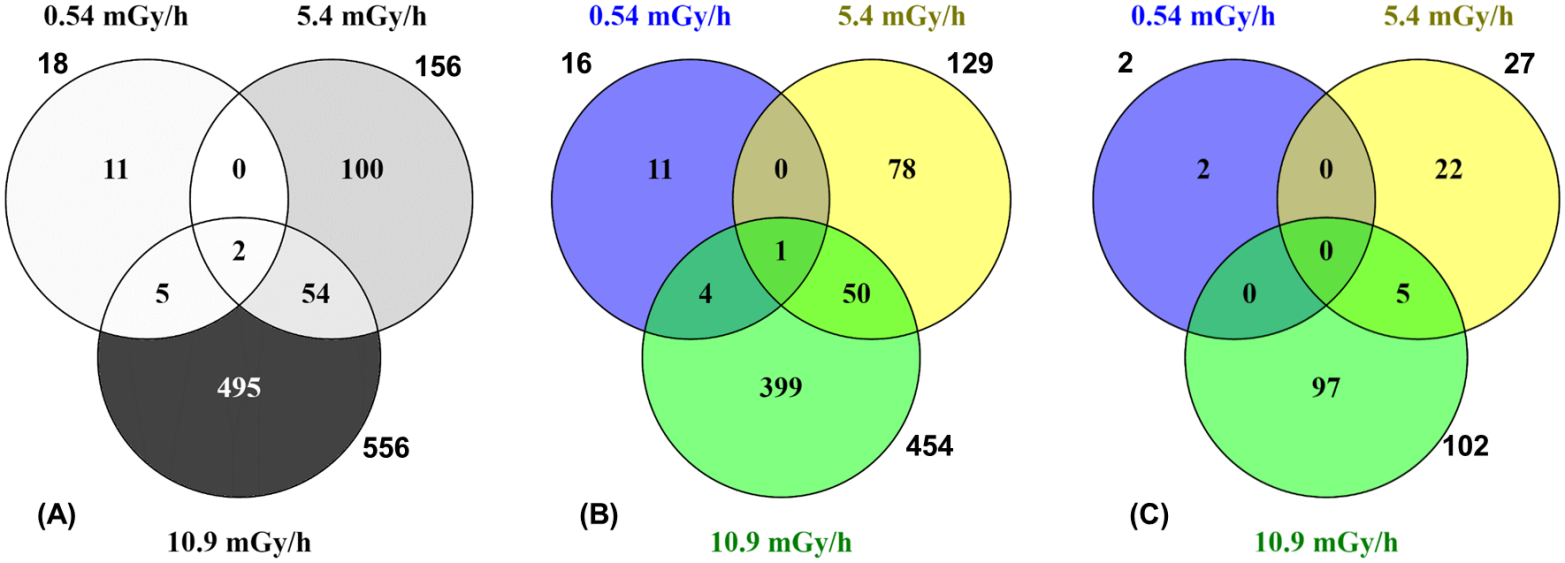
Venn diagram showing common and unique sets of differentially expressed genes between exposure treatments. Total number of **(A)** common genes between 0.54, 5.4 and 10.9 mGy/h after pairwise comparison to controls (FC ± 1.3, FDR < 0.05); **(B)** Up-regulated genes; **(C)** Down-regulated genes.

In the 0.54 mGy/h exposure group, 16 genes were up-regulated (FC from 1.3 to 2.2) and two genes down-regulated with FC from 1.3 to 1.7 (Fig 2, S4 Table). In the 5.4 mGy/h exposure group, 129 genes were up-regulated with FCs from 1.3 to 674, while 27 were down-regulated with FCs from 1.3 to 2 (Fig 2, S4 Table). In the 10.9 mGy/h exposure group 556 DEGs were split between 454 up-regulated with FCs from 1.3 to 3.2 and 102 down-regulated genes with FCs of 1.3 to 2.4 (Fig 2, S4 Table). Among the DEGs, two were found to be differentially expressed in all three exposure groups: *pfkfb3* (6-phosphofructo-2-kinase-fructose-2,6-biphosphatase 3) up-regulated in 0.54, but down-regulated in the 5.4 and 10.9 mGy/h; *crabp2b* (cellular retinoic acid binding protein 2b) which is similarly up-regulated in all exposure groups (Fig 3A, S4 Table).

**Fig 4:**
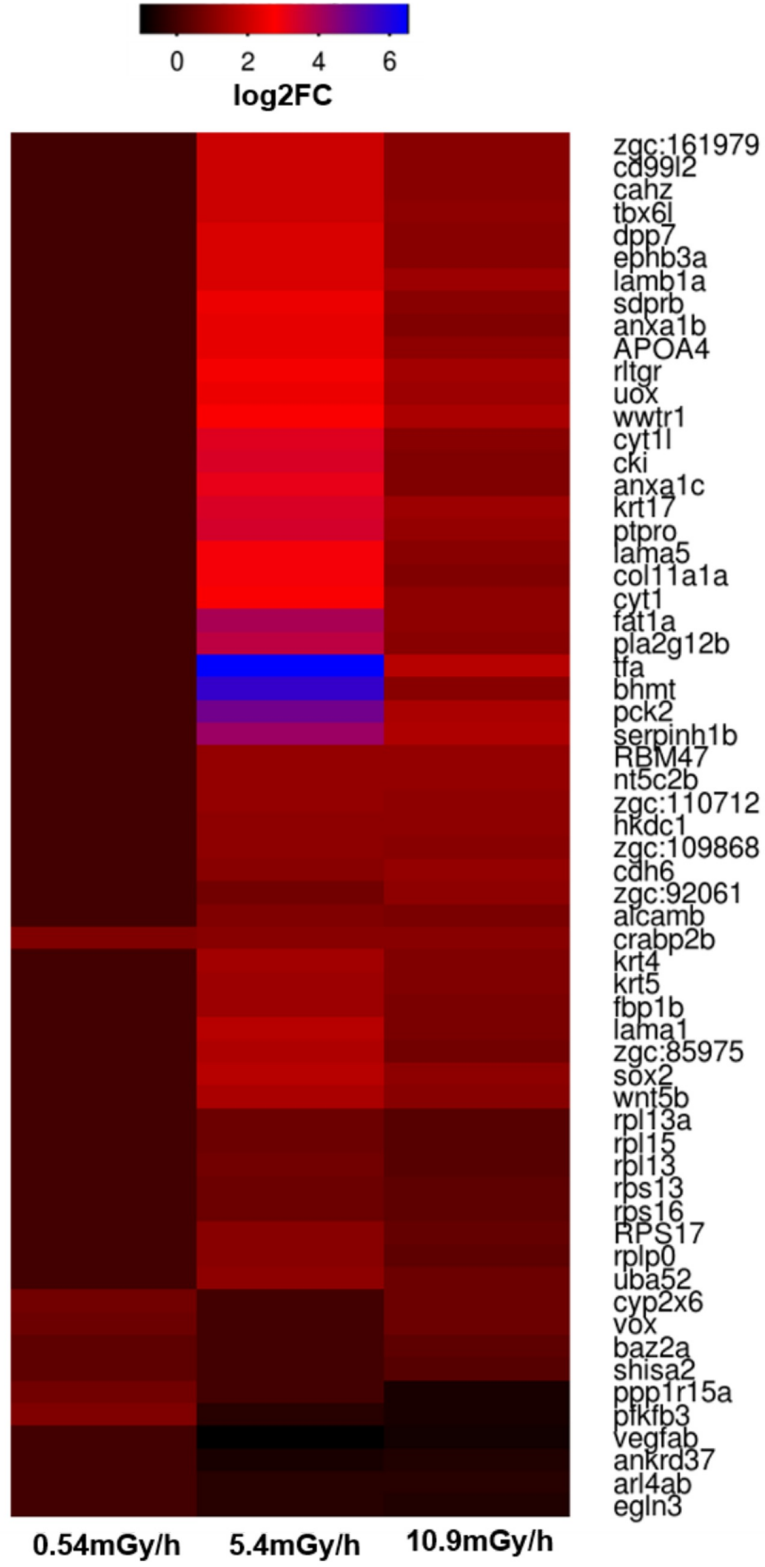
Expression levels for up and down-regulated overlapping genes between exposures. Expression levels in the 0.54, 5.4 and 10.9 mGy/h groups are given in log2 of the fold changes (FC).

In addition, five and 54 DEGs were overlapping between the 0.54/10.9 and 5.4/10.9 mGy/h groups, respectively (Fig 3A). As for the up-regulated DEGs, four genes were overlapping between the 0.54 and 10.9 mGy/h, while 50 genes were overlapping between the 5.4 and 10.9 mGy/h group (Fig 3B). Furthermore, down-regulated overlapping genes were found (five genes) only between the 5.4 and 10.9 mGy/h exposure (Fig 3C). The most up-regulated common gene in the 10.9 mGy/h group, *tfa* (transferrin-a), was also highly up-regulated in the 5.4 mGy/h group (S4 Table), although the FC values differed between the groups (S4 Fig). In addition, lipoprotein genes: *apoBb* (apolipoprotein Bb), *apoA1a* and *apoA1b* (apolipoprotein A-Ia/Ib), and common with the 10.9 mGy/h group, *apoA-IV* (apolipoprotein A-IV) were amid the top up-regulated in the 5.4 mGy/h group. The most down-regulated common gene between 5.4 and 10.9 mGy/h was *vegfab* (vascular endothelial factor Ab (S4 Table). The expression levels for up and down-regulated genes overlapping between the three dose rates are presented in Fig 4.

### Pathway analysis

#### General pathways analysis

**Fig 5:**
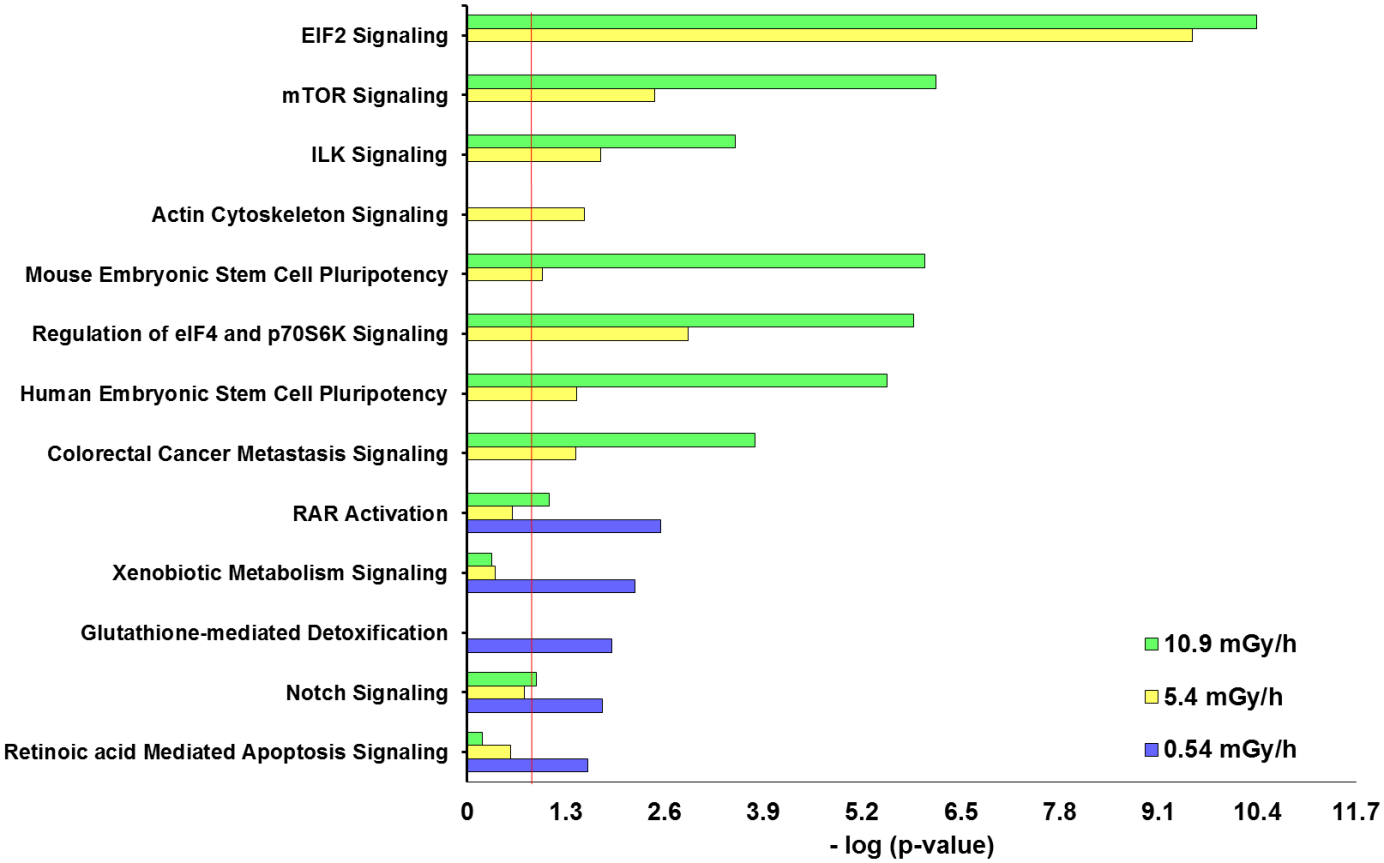
DEG functional analysis (IPA). Top signaling pathways in canonical pathway comparison between all exposure groups: 0.54 mGy/h, 5.4 mGy/h and 10.9 mGy/h. The rankings were based on Fisher's exact test and high-ranking categories are displayed along in a decreasing order of significance from top. The threshold line (red) denotes the cut-off for significance (p-value 0.05).

The core analysis IPA software tool was used to find the most significantly affected biological signaling (canonical) pathways by the DEGs in the three exposure groups. A statistically significant difference between the signaling pathways in the 0.54 mGy/h exposure group compared to the 5.4 and 10.9 mGy/h was found. In the lowest dose rate, the most affected signaling pathway was retinoic acid receptor activation *(RARa)*, followed by *RA* mediated apoptosis and glutathione mediated detoxification signaling (Fig 5).

Interestingly, compared to the signaling pathways in 0.54 mGy/h, the higher doses demonstrated some *RA* pathway activity, but this was below the significance threshold (Fig 5, S11 Fig). In the two higher dose rates, the most significantly affected signaling pathways were *eif2* (eukaryotic initiation factor 2) and *mTOR* (mechanistic target of rapamycin), which were not affected (p-value > 0.05) in the lowest dose rate group (Fig 5, S12 and S13 Figs).

#### Toxicological pathways

**Fig 6:**
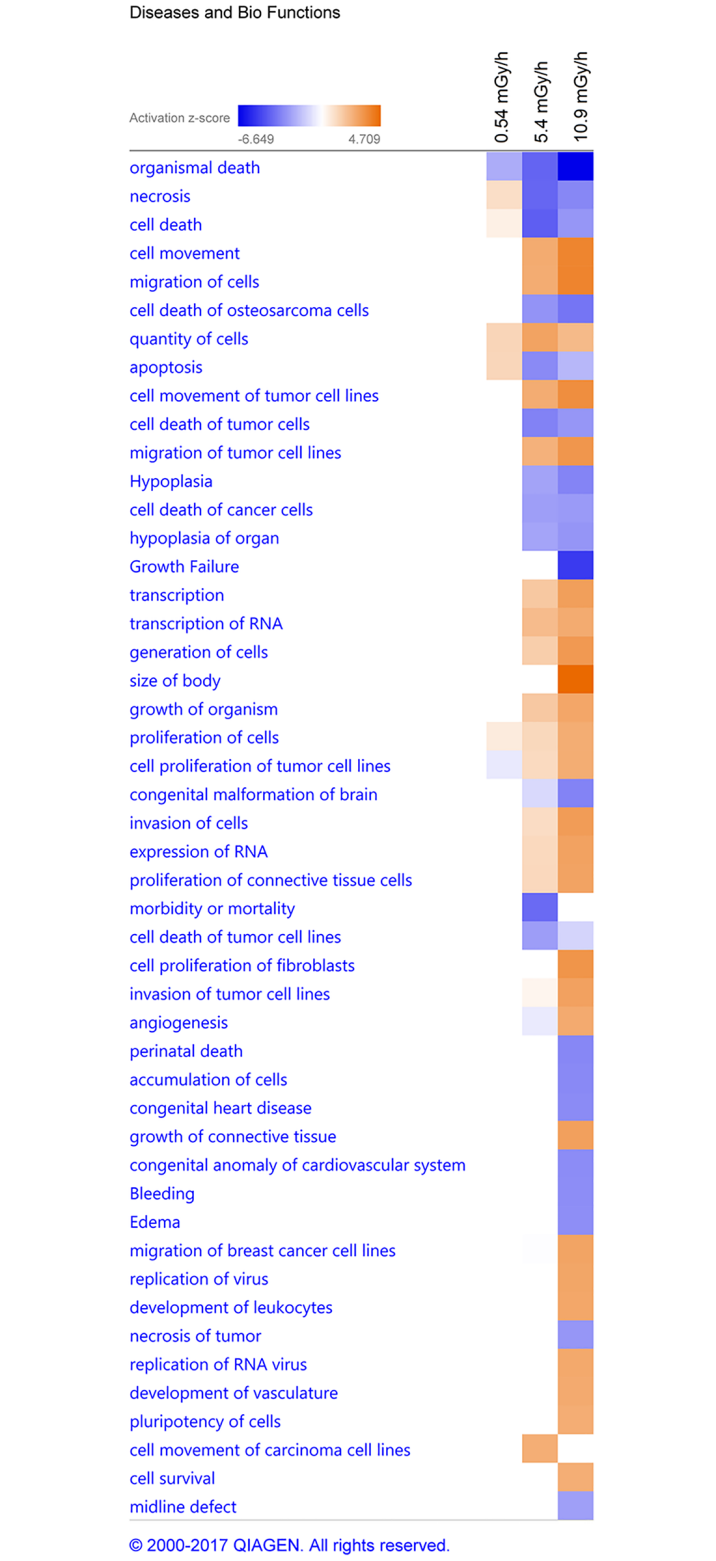
The most affected biological function and diseases networks. IPA predictions in comparison analysis between 0.54 (1) 5.4 (2) and 10.9 mGy/h (3) group. The heat map is based on the activation z-score, consistent with the particular activation state: “activated” (orange) or “inhibited” (blue). Z-score cut off set to ± 2.5 (arbitrary).

To identify the top diseases and biological functions of altered genes in each exposure group, the gene expression data sets were compared between all exposure groups in IPA. The DEGs in the datasets were shown to be involved in gene networks associated with various embryonic developmental processes and cell functions (Fig 6).

In the 0.54 mGy/h exposure group, gene networks associated with apoptosis and other cell death mechanisms were active, while gene networks associated with organismal death and proliferation of tumor cell lines (Fig 6) were inhibited. In contrast, in the 5.4 and 10.9 mGy/h groups, gene networks associated with apoptosis were inhibited and gene networks related to proliferation of tumor cell lines were active. Similarly to the lower dose rate exposure, the gene networks related to organismal death in these groups were inhibited. Comparison of expression of apoptosis genes showed that of total 129 DEGs found in the network, 5 were found in the 0.54 mGy/h group (all up-regulated), 40 in the 5.4 mGy/h group (34 up- and 6 down-regulated) and 101 in the 10.9 mGy/h (83 up- and 18 down-regulated) (S14 Fig). The one common and similarly expressed gene between all exposures in the apoptosis network was *crabp2b*, while expression levels of 16 common genes between 5.4 and 10.9 mG/h groups differed (S14 Fig). Additionally, networks associated with cell movement, growth, cardiovascular developmental processes and cancer development were significantly activated in the two higher dose rate exposure groups; albeit more significantly in the highest dose (Fig 6).

#### Key regulators

A transcription factor enrichment analysis was conducted to identify upstream regulators of transcriptional networks modulated by ionizing radiation. A total of 159, 632 and 939 transcription regulators in the 0.54, 5.4 and 10.9 mGy/h exposures were identified, respectively (S5 Table). *Myc* (v-myc avian myelocytomatosis viral oncogene derived homolog), *TNF* (tumor necrosis factor), *tp53* (tumor protein p53) and *hnf4a* (hepatic nuclear factor 4, alpha) were identified as upstream regulator genes in all exposure groups (S5 Table, S5–S8 Figs). In the two higher dose rates, *TGFb1* (transforming growth factor, beta 1) and *cebpa* (CCAAT/enhancer binding protein C/EBP, subunit alpha) were found to be significant upstream regulators (S5 Table, S9 and S10 Figs).

### Validation by quantitative real-time PCR (qPCR)

In order to validate the RNA sequencing results, eight differently expressed genes were selected for quantitative real-time polymerase chain reaction (qPCR) analyses in the groups exposed to 0.54, 5.4, 10.9 mGy/h and controls. The selected genes and their respective fold changes are presented in Table 3. The data from real-time qPCR and the RNA-sequencing showed a good correlation (Pearson’s linear correlation coefficient r = 0.89, p < 0.0001).

**Table 3:** Real time qPCR verification of RNA sequencing. Results presented as fold change (FC) for eight genes. The (n.a.*) refers to not differentially expressed, while the fold change was not available for this gene in this group.

Gene ID		FC RNA-seq			FC RT qPCR		
	Dose rate (mGy/h)	0.54	5.4	10.9	0.54	5.4	10.9
pfkfb3		1.8	1.3	1.5	1.8	1.4	0.9
crabp2b		1.9	2.1	2.0	2.9	2.3	2.0
vox		1.5	1	1.5	1.6	2.2	0.7
ppp1r15a		1.6	0.8	1.5	1.8	1.3	0.6
shisa2		1.3	1	1.3	1.5	1.9	0.7
sox2		n.a.*	3.3	2.2	3.0	1.6	0.8
tfa		n.a.*	93.2	3.2	1.2	40.7	20.7
eef2b		1.1	1.3	1.2	0.6	1.6	1.4

Two of the selected genes are common between all three exposure groups (*pfkfb3* and *crabp2b*). Three are common between 0.54 and 10.9 mGy/h groups (*vox*, *ppp1r15a* and *shisa2*) and between 5.4 and 10.9 mGy/h (*sox2*, *tfa* and *eef2b*). Only two genes were found to have an opposite regulation at one of the dose rates; *pfkfb3* in the 5.4 mGy/h group was up-regulated, while *shisa2* in the 10.9 mGy/h was down-regulated (Table 3).

## Discussion

Previous studies in zebrafish reported underlying molecular mechanisms responsible for adverse biological effects such as DNA damage [28,29], ROS, oxidative stress, apoptosis, bystander effects [30–32] and also genetic [32–34] and epigenetic changes [8] following exposure to ionizing radiation. However, most of the genetic responses were studied following acute exposures.

In this study, we focused on potential adverse effects on the embryonic development caused by low dose and dose rate ionizing radiation. To this end, we investigated the developmental and toxicological effects of continuous gamma irradiation (doses between 17.5–3496 mGy) during early blastula (2.5 hpf; 256-cell stage), through to the hatching period (48–72 hpf) and early larval development, i.e., life stages associated with numerous delicate morphological changes [35].

To investigate molecular initial events associated to effects of ionizing radiation later in development, analysis of the gastrula stage 5.5 hpf embryo transcriptome was carried out using RNA sequencing combined with a functional gene network analysis software.

### Adverse effects of radiation in developing embryos

The results from the observations of survival, deformities and total hatching at 96 hpf showed that radiation caused a significant dose-dependent reduced survival, affected the total hatching and increased the number of deformities. (Table 2, Fig 1). The exposure dose rates for evaluating the phenotypic effects used in the present work (0.4, 3.9, 15, and 38 mGy/h) were higher than the ERICA screening value of 10 μGy/h (0.24 mGy/d) [10]. However, the dose-rates span the proposed level of 0.42 mGy/h (10 mGy/day), which is considered to be a level below which there is not likely to be any detrimental effect on aquatic populations (UNSCEAR Report, 1996) and the derived consideration reference levels (DCRL) for fish (~0.42 mGy/h– 40 mGy/h), at which there are “likely to be some observable adverse effects occurring to individuals” [36].

The lowest dose rate in the present work at which deformities were observed was 0.4 mGy/h (total dose 36.8 mGy). The onset of hatching was premature in the 0.4 mGy/h exposure group (17.5 mGy total dose, Table 1), and significantly delayed in the 38 mGy/h group (1664 mGy total) (S2 Table). The total hatching in these groups was unaffected (Table 2). Interestingly, in a previous study of hatching intervals following X-ray exposure during the blastula stage, earlier hatching was associated with low dose (25 mGy at 0.43 Gy/min), while higher doses (250–500 mGy) delayed the onset of hatching [37]. In addition, other studies report that both low and high doses had an accelerating effect on the hatching interval [28,32]. In the 15 mGy/h group, the total hatching was significantly decreased (Table 2). A similar result was reported after X-rays exposure to 500 mGy [37], which is close to the total dose (657 mGy) in the present 15 mGy/h exposure group (Table 1). The survival, although exceeding 82% in all groups (S2 Table), was significantly lower than control in the 38 mGy/h group (Table 2) after both 43.8 and 92 hours of exposure. Previously, mortality in zebrafish embryos was reported only for acute exposures from 1 to 24 hpf (1–10 Gy, X-rays) [38]. Although embryo mortality from the 43.8 h exposure was observed at 48 hpf, no further increase was observed at 96 hpf (S2 Table). Collectively, these observations might indicate that the early developmental stages, prior to the hatching interval, are more sensitive to the effects of ionizing radiation, resulting in mortality. Other studies have reported no significant differences in embryo viability after receiving acute ionizing radiation doses ranging from 1–10 Gy [34,39], nor following continuous exposures up to 24 mGy/h (2280 mGy) [32], although the latter induced multiple deformities. Generally, the adverse effects on embryo development from the continuous exposure in the present study showed considerable variability in response to lower and higher doses, and in order to elucidate potential molecular mechanisms behind the observed effects, this variability was further studied by transcriptomics.

### The 5.5 hours post fertilization embryo transcriptome

The gene expression analysis was performed at the late blastula / early gastrula stage (~ 5.5 hpf), a critical stage of embryogenesis, characterized by intensive cell proliferation and specification [17,35]. At this stage the zygotic genome is activated, while the inherited maternal transcript (synthesized during oogenesis and stored in the egg) is degraded [15]. Thus, changes in transcriptome profile can be attributed to radiation effects on the transcriptional program of the embryo’s own genome. The choice of this stage served two major aims: early toxic effects and accompanying stress or defense mechanisms would be reflected at the transcriptional level, and deviation of the transcriptional program at this stage could be indicative or predictive to adverse outcome observed later during embryogenesis. The applied dose rates were selected to both encompass a toxic effects dose response and to be environmentally relevant. The RNAseq analysis was thus conducted on low total doses, which consequently should produce only low level of DNA damage. This strategy enables investigation of more subtle and less well-described molecular effects of ionizing radiation in addition to genotoxicity. The fact that significant transcriptional changes could be observed from a 3 hour exposure to total doses from 1.6 to 33 mGy corroborates the validity of the approach. Moreover, the observed responses were intelligibly connected to the adverse outcomes observed at the phenotype level. This correlation is important with respect to the level of dose rates and total doses that would be required to elicit changes at the molecular level.

The number of similarly and differently expressed genes, as well as overlapping DEGs, showed a clear dose-response effect in the gamma exposed embryos with the lowest number of modulated genes in the 0.54 mGy/h group and with an increasing number in the two highest exposure groups (5.4 and 10.9 mGy/h) (Fig 2). A considerable variation in FC between the 5.4 and 10.9 mGy/h groups was observed (S4 Fig), but a total of 56 DEGs were common in these exposure groups.

Two genes, *pfkfb3* and *crabp2b*, were found to be differentially expressed in all exposures. The *pfkfb3* gene is involved in regulating the expression of cyclin-dependent kinase 1, which promotes proliferation and survival in tumor cells [40] by protecting cancer cells against oxidative stress through S-glutathionylation and glucose metabolism switch to the pentose phosphate pathway [41,42], and thereby counteracting ionizing radiation generated ROS. The *crabp2b* gene is a one of the two zebrafish *crabp2* genes orthologous known to encode retinoic acid (*RA*) protein family and lipocalin/cytosolic fatty acid binding protein family. Interestingly, the *crabp2b* was found to be similarly up-regulated (FC ~ 2) in all three irradiation treatments in both the RNA-seq and the qPCR (Table 3, Fig 4). Retinoic acid is the biological active metabolite of Vitamin A and *crabp2* regulates the access of retinoic acid by binding with the nuclear retinoic acid receptor alpha (*RARa*) [43] and helps modulating the RA gradient, which is important for the development of vertebrates, including humans [43,44]. Deficient or excess levels of vitamin A have induced malformations in experimental animals and humans, indicating that the concentration must be kept within a narrow range [45,46]. Furthermore, *crabp2b* is associated with regulation of the hindbrain anterior-posterior axis development [47], and is expressed in structures requiring the retinoic acid during embryonic development, such as the CNS, dorsal retina, branchial arches, epidermis, otic vesicle and pectoral fins [43]. Considering the increased number of malformations observed in irradiated fish, it could be hypothesized that this is in part induced by modification of the *crabp2b* gene.

Among the common genes modulated by 5.4 and 10.9 mGy/h exposures, the most significantly up-regulated gene is *tfa* (Fig 4). This gene is critical for iron transport and iron regulated hormone expression [48], and is involved in the immune response to bacterial infection [49]. A decrease in concentration of the transferrin protein was found in blood plasma of radiological accident victims compared to blood plasma from non-irradiated individuals, and reported as a possible mutagenic factor [50]. However, a protective role of the transferrin pathway for antioxidant repair and sequestering metals was also suggested [51]. Additionally, increased chromosomal damage combined with increased transferrin was demonstrated in lymphocyte cultures following exposure to 1 Gy of ionizing radiation, suggesting that transferrin is affected by radiation [52].

The highly up-regulated apolipoprotein genes in the two higher dose rate exposure groups, and notably the 5.4 mGy/h group (*apoBb*, *apoA1a*, *apoA1b* and *apoA-IV*), could point to radiation affecting mechanisms behind the lipid metabolism and transport from yolk cells to the embryo (S4 Table) [53]. Apolipoprotein genes play a role in reducing fat intake during embryonic development, as previously shown in zebrafish [53] and humans [54], causing malnutrition of the embryo, which may have disrupted normal development. In addition, apolipoprotein genes were reported to negatively regulate (*apoB*) [55], or even inhibit the angiogenesis (*apoA1*), in a *vegf* down-regulation dependent pathway [56]. Relatedly, among the common genes modulated by 5.4 and the 10.9 mGy/h treatments, the most significantly down-regulated gene in both data sets is *vegfab* (FC 1.6–2), an isoform of the human ortholog *VEGF-A* (Fig 4) [57]. At early life stages, this gene mediates differentiation of endothelial cells and early vascular development and angiogenesis (formation of new blood vessels) [58], including retinal angiogenesis [59]. In developed individuals, *vegf* stimulates the angiogenesis [60], either in a physiological (such as tissue repair processes) or pathological states (such as tumor growth), and *vegf* activity has been shown to be stimulated through an intracellular increase in ROS generated as a result of exposure to ionizing radiation [61]. In an experimental study of radiation effects in mice, *vegf* together with *eif2* was modulated in bladder tissue [62].

### Molecular pathways—Potential mechanisms of radiotoxicity

A transcription factor enrichment analysis was performed to investigate whether gamma induced pathways or gene networks could be ascribed to master regulators. IPA analyzes of the datasets identified upstream regulator genes, which were not necessarily significantly affected, but may play key roles in the regulation of DEGs. The transcription factors *myc*, *TNF*, *tp53* and *hnf4a* were found to be in central positions of functional networks of modulated genes in comparison between the three exposure groups (S5 Table). Additionally, *TGFb1* and *cebpa* were identified as key regulators at the two higher dose-rates (5.4 and 10.9 mGy/h) (S5 Table).

*Myc* was found to be one of the top upstream regulators, in all three exposures (S5 Table, S5 Fig) and is implicated in the regulation of various processes in the cell, such as growth and proliferation, migration, differentiation and cell death. Up-regulation of the oncogenes *myc* and *mycn* is associated with poor outcomes of several cancers, such as aggressive neuroblastoma [63], large B cell lymphoma [64], acute myeloid leukemia (AML) [65] and nephroblastoma (Wilms tumor) [66]. Combined *Myc* up-regulation with an altered retinoic acid (*RA*) pathway activity worsens the prognosis of such cancers [67]. Furthermore, *TNF* was found to regulate a high number of molecules in the datasets (S5 Table, S6 Fig). This cytokine was previously shown to be strongly protective at lower ionizing radiation doses for the hematopoietic stem cell system [68] and via selective destruction of blood vessels in T-cell tumors [69]. Interestingly, the activity of tumor necrosis factor-alpha (*TNF-α*) in cell lines was found to be antagonistic to the activity of *TGFb* [70]. Another identified upstream regulator, *tp53* (S5 Table, S7 Fig), is known to regulate apoptosis in response to DNA damage [71], but was also demonstrated to be a critical factor for normal development and survival in zebrafish embryos after exposure to ionizing radiation [72,73]. *Tp53* was found to decrease, but also to concomitantly regulate tumor suppressive *TGFb* responses through Smad2/3 DNA complexes [74]. Although not differentially expressed in the 0.54 and 5.4 mGy/h datasets, *hnf4a* is found to be a transcription regulator for a large number of DEGs in all exposure groups (S3 Table, S8 Fig). This transcription regulator was found to be up-regulated in the blood of patients exposed to ionizing radiation [75], and in a human tissue model exposed to low dose gamma radiation [76]. *Hnf4a* regulates the gastrulation [77], the developmental period during which the morphogenetic cell movements, along with production of the three primary germ layers (ectoderm, mesoderm and endoderm) and the embryonic axis (> 5.25 hpf) occur [78]. It is mainly expressed in the digestive system and in the brain. This data propose *hnf4a* as a factor involved in the induction of biological effects of radiation in humans as well as in other vertebrate species.

An activated predicted upstream regulator in both the 5.4 and 10.9 mGy/h, but not in the 0.54 mGy/h exposure was *TGFb1* (S3 Table, S9 Fig). The *TGFb1* cytokine regulates a variety of functions, and is known to be a mediator of the apoptosis, redox homeostasis and bystander effects in tissues and cells in response to radiation [69,79–81]. In addition, *TGFb* was found to co-regulate angiogenesis in tumors with *vegf* [82]. IPA also identified *cebpa* as a regulator gene among the common DEG in the two higher dose rates (5.4 and 10.9 mGy/h) (S10 Fig). In the study of B-cell chronic lymphocytic leukemia (B-CLL) patients in the post-Chernobyl period, similar key regulator genes, gene networks and signaling pathways were altered [83]. *Cebpa* is associated with regulation of hematopoiesis, hematopoietic stem cell migration, liver development and regulation of transcription [84]. It is predominantly found in mature myeloid cells and is required for the differentiation of myeloid cells in order to prevent the occurrence of myeloproliferative diseases [85]. Diseases associated with a down-regulation of *cebpa* include acute myeloid leukemia with *cebpa* somatic mutations [86]. Moreover, other studies have reported that ionizing radiation caused increased expression of *cebpa*, which was associated with a reduction of hematopoietic stem cells and the self-renewal of multipotent hematopoietic progenitor cells [87]. The similar regulation of these genes in mammals and zebrafish may suggest that similar mechanisms might be behind the molecular changes following exposure to radiation.

The signaling pathways affected most significantly by the 0.54 mGy/h exposure (*RAR* activation, *RA* mediated apoptosis and glutathione mediated detoxification seem to be consistent with the described repair mechanisms occurring at low doses. This adaptive response to low doses of ionizing radiation in biological systems is mainly characterized by antioxidant mediated detoxification of ROS, more rapid DNA repair, apoptosis signaling and stimulated immune response [88,89].

In the 5.4 and 10.9 mGy/h treatments, *eif2* and *mTOR* were the most significantly up-regulated signaling pathways. A significant role of the *eif2* signaling pathway is the adaptive response to stress by regulating the formation of translation initiation complexes, which leads to reduced recognition of AUG start codons and therefore total translational inhibition and the induction of apoptosis [90]. The *mTOR* (mammalian target of rapamycin) signaling pathway is centrally involved in cell metabolism, growth, proliferation and survival via regulation of protein synthesis and mRNA stabilization [91]. Furthermore, it is activated during tumor formation and modulation of angiogenesis, development of diabetic retinopathy [92] and in radiation induced apoptosis [93]. A dysregulation of *mTOR* was reported to affect the premature aging of cells and destabilize the cytoskeletal structure after exposure to chronic ionizing radiation, in addition to changes in the *eif2* signaling pathway [94]. The *eif2* signaling pathway was in comparison to the present results found to be down-regulated in the blood of post Chernobyl leukemia patients [83]. The predicted top diseases and biological functions (IPA), suggest that the changes in signaling pathways and gene expression in the lower dose-rate (0.54 mGy/h) are activating gene networks associated with apoptosis and other cell death mechanisms in the embryos, while inhibiting proliferation of tumor cell lines (Fig 6). In the higher dose-rate exposure groups (5.4 and 10.9 mGy/h), gene networks involved in cell death and apoptosis were shown to be inhibited, while cell movement, cardiovascular development and tumor development were activated (Fig 6). The predictions from the gene expression suggest an early response of the developing embryos to continuous ionizing radiation and would be interesting to address in follow up studies using genetic, epigenetic and mutagenesis methods.

## Conclusion

Continuous exposure to external gamma radiation at environmentally relevant dose-rates (from 0.4 mGy/h, total dose 17.5 mGy) resulted in severe consequences for the development and gene expression of zebrafish embryos and larva. Significant mortality compared to controls was observed in the groups exposed to the highest dose rate (38 mGy/h), while increased number of deformities and differences in the hatching was observed in groups exposed to lower doses ≥ 0.4 mGy/h (Tables 1 and 2 and S2 Table). Consistent with the observed adverse effects, the changes in gene transcription could be attributed to cell differentiation and morphological development. The results suggest that active repair mechanisms mediated by antioxidants could be the reason for the lack of phenotypic observable effects in the lower dose. The higher radiation dose rates instigate, among others, genes and networks involved in cell cycle control (tp53), translation and cell survival (eif2, mTOR), and disrupted development and cancer (myc, TGFb1, hnf4a, cebpa), which in sum increase the risk for an adverse effect. Thus, RNA sequencing enabled identification of molecular initiating events from a 3 hour gamma radiation exposure to 0.54, 5.4 and 10.9 mGy/h (total dose 1.6 to 33 mGy), which are consistent with the phenotype level adverse outcomes observed in 96 hpf stage larvae.

## Supporting information

S1 Table Real time qPCR primers. (XLSX)

S2 Table Survival and hatching. Survival and hatching after 43.8 and 92 hours exposure to specified dose rates. Survival at the 43.8 hours exposure did not differ at 48 and 96 hpf. All values presented as mean percentage ± 95% confidence interval (CI).
(XLSX)

S3 Table Mapping statistics. Mapping statistics presented separately for each replicate (A) and each exposure (B) with their respective controls. Approximately 60% of the reads were mapped to the reference genome. Of the mapped reads, ~ 40% were mapped when allowing no mismatch, while ~ 15% of the reads were mapped when ≤ 2 bp mismatches were allowed. On the other hand, ~ 56% out of the mapped reads were found to represent unique genome positions, with ~ 1.5% of reads mapping to multiple positions.
(XLSX)

S4 Table Full DEGs list. (XLSX)

S5 Table IPA upstream regulators. (XLSX)

S1 Fig Bioinformatic analysis pipeline. (TIF)

S2 Fig RNA-seq mapping frequency of reads distribution. Differential expression threshold is FC ± 1.3. **A**, **B**, **C** and **D** show the distribution of mapped reads at 0.54, 5.4 and 10.9 mGy/h. **E** and **F** represent the distribution of mapped reads in control groups for the lowest (0.54 mGy/h) and for higher dose rates 5.4 and 10.9 mGy/h, respectively. All libraries were mapped to the ZF genome (Zv9).
(TIF)

S3 Fig Multidimensional scaling (MDS) plot of RNA-seq libraries after trimmed mean of M-values (TMM) normalization. **A)** Group exposed at 0.54 mGy/h and the control group for the lowest dose. Two and three biological replicates of the exposed group and controls, respectively, were included in the analysis. **B)** and **C)** Groups exposed to 5.4 and 10.9 mGy/h and controls. Three replicates were included.
(TIF)

S4 Fig Principal component analysis (PCA) of gene expression data. Analysis was conducted by pairwise comparison of exposed and their respective controls. **A)** 0.54 mGy/h, **B)** 5.4 mGy/h and **C)** 10.9 mGy/h. Expression values were log2 transformed. Black and red dots represent non-differential and differentially expressed genes respectively (FDR < 0.05) (edgeR v3.4.2 Bioconductor).
(TIF)

S5 Fig *Myc* upstream regulator (IPA). *Myc* target gene networks and interactions, presented in a subcellular layout as part of the 10.9 mGy/h group.
(TIF)

S6 Fig *TNF* upstream regulator (IPA). *TNF* target gene networks and interactions, presented in a subcellular layout as part of the 10.9 mGy/h group.
(TIF)

S7 Fig *Tp53* upstream regulator (IPA). *Tp53* target gene networks and interactions, presented in a subcellular layout as part of the 10.9 mGy/h group.
(TIF)

S8 Fig *Hnf4a* upstream regulator (IPA). *Hnf4a* target gene networks and interactions, presented in a subcellular layout as part of the 10.9 mGy/h group.
(TIF)

S9 Fig *TGFb1* upstream regulator (IPA). *TGFB1* target gene networks and interactions, presented in a subcellular layout as part of the 10.9 mGy/h group.
(TIF)

S10 Fig *Cebpa* upstream regulator (IPA). *Cebpa* target gene networks and interactions, presented in a subcellular layout as part of the 10.9 mGy/h group.
(TIF)

S11 Fig Gene expression in *RARa* pathway (IPA). (TIF)

S12 Fig Gene expression in *eif2* pathway (IPA). (TIF)

S13 Fig Gene expression between in *mTOR* pathway (IPA). (TIF)

S14 Fig Gene expression in apoptosis network (IPA). (TIF)

S15 Fig Deformities in zebrafish larva exposed to gamma radiation. The observations were done at 96 hours post fertilization (hpf). **A.** Control larva showing normal development; **B-C.** Larvae exposed to 38 mGy/h for 92 hours (Group “B”), demonstrating general developmental defects and short-tails.
(TIF)
