# Supplementary figures and images for: Dose-dependent effects of gamma radiation on the early zebrafish development and gene expression
Source: PLoS One. 2017 Jun 19;12(6):e0179259. doi: 10.1371/journal.pone.0179259 (PMC5476279; doi:10.1371/journal.pone.0179259)

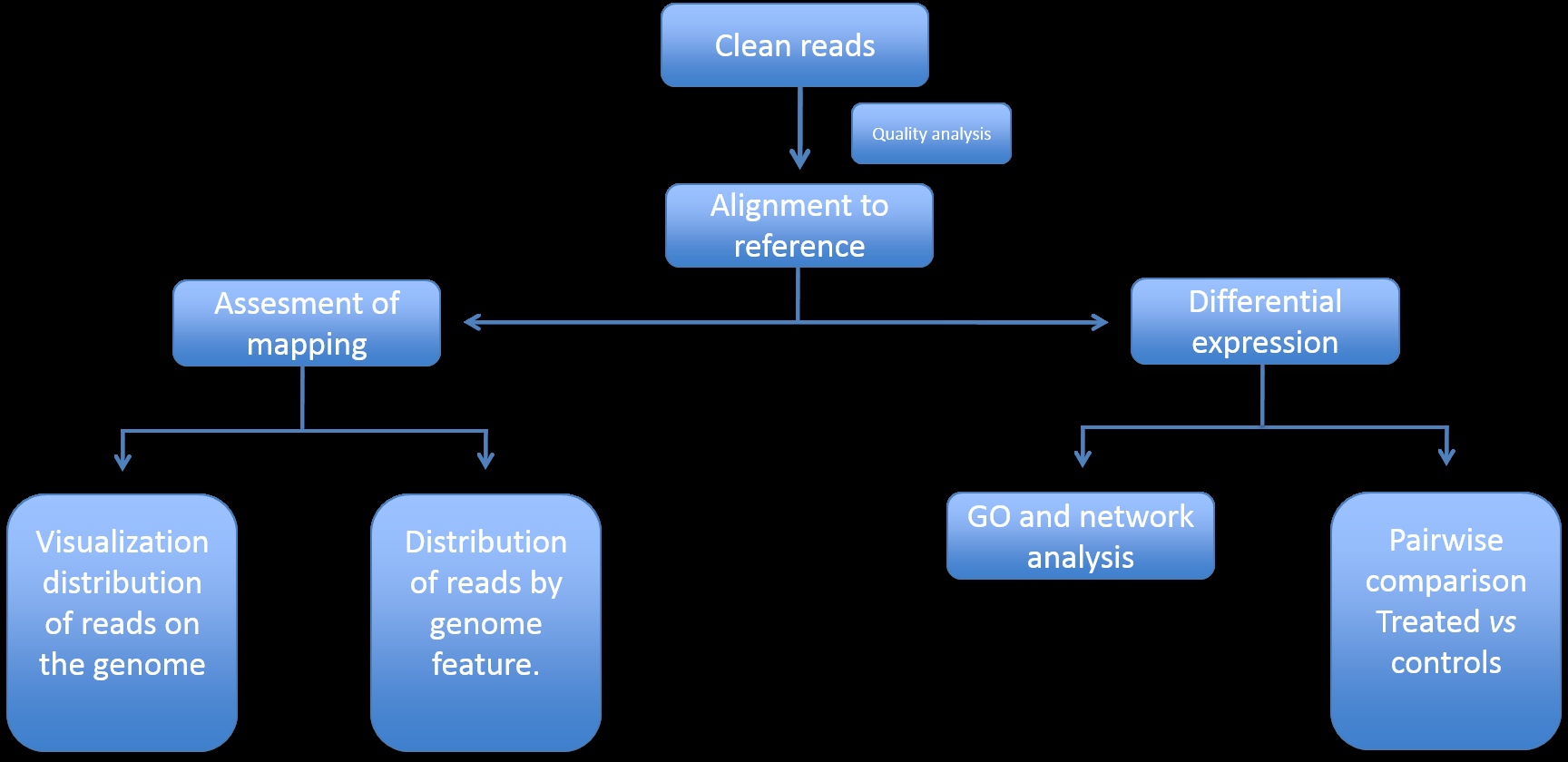

Supplement: S1 Fig — (TIF) [file pone.0179259.s006.tif]

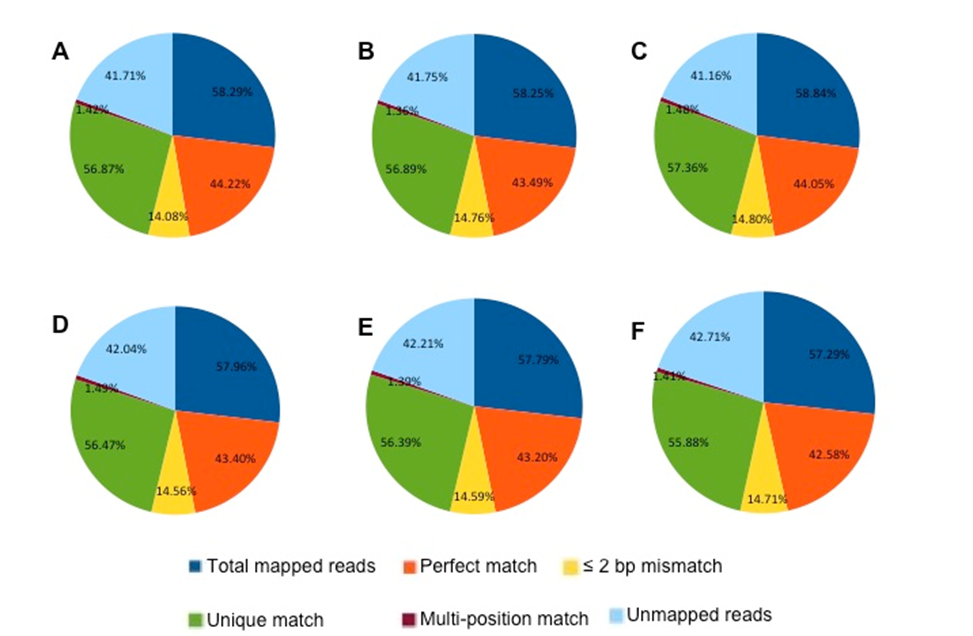

Supplement: S2 Fig — Differential expression threshold is FC ± 1.3. A, B, C and D show the distribution of mapped reads at 0.54, 5.4 and 10.9 mGy/h. E and F represent the distribution of mapped reads in control groups for the lowest (0.54 mGy/h) and for higher dose rates 5.4 and 10.9 mGy/h, respectively. All libraries were mapped to the ZF genome (Zv9). (TIF) [file pone.0179259.s007.tif]

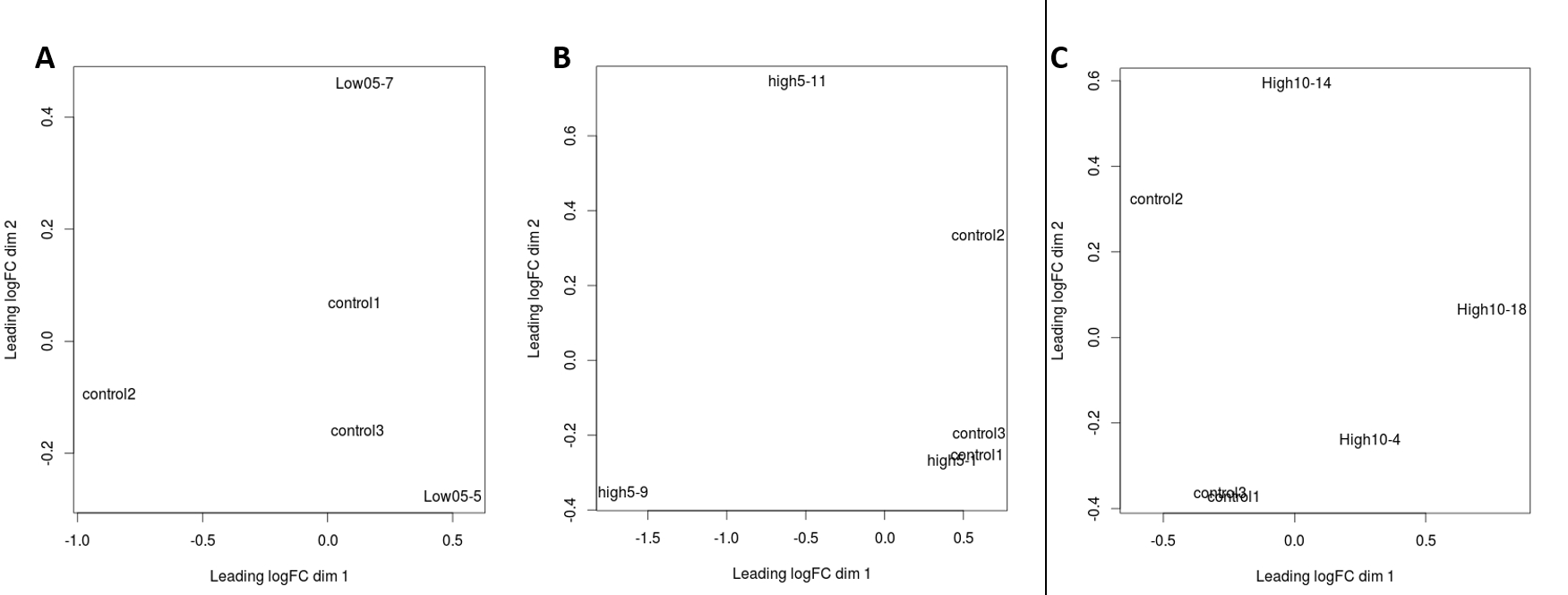

Supplement: S3 Fig — A) Group exposed at 0.54 mGy/h and the control group for the lowest dose. Two and three biological replicates of the exposed group and controls, respectively, were included in the analysis. B) and C) Groups exposed to 5.4 and 10.9 mGy/h and controls. Three replicates were included. (TIF) [file pone.0179259.s008.tif]

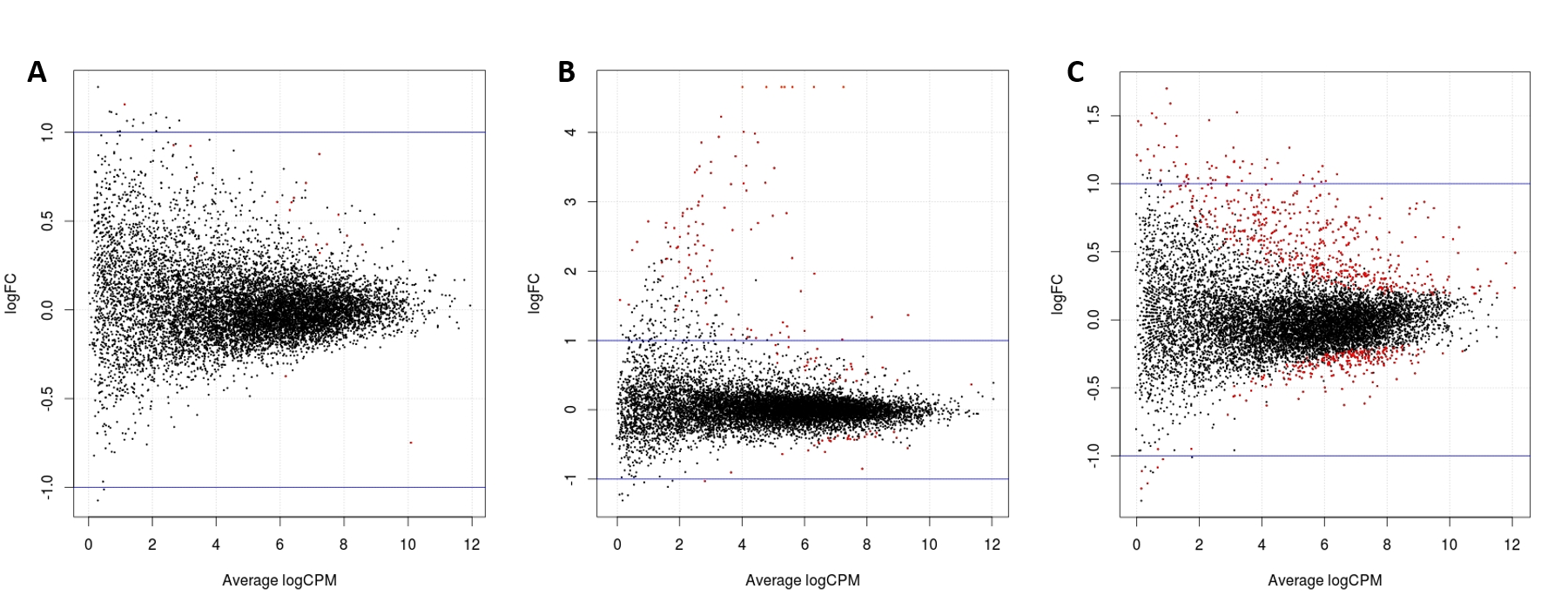

Supplement: S4 Fig — Analysis was conducted by pairwise comparison of exposed and their respective controls. A) 0.54 mGy/h, B) 5.4 mGy/h and C) 10.9 mGy/h. Expression values were log2 transformed. Black and red dots represent non-differential and differentially expressed genes respectively (FDR < 0.05) (edgeR v3.4.2 Bioconductor). (TIF) [file pone.0179259.s009.tif]

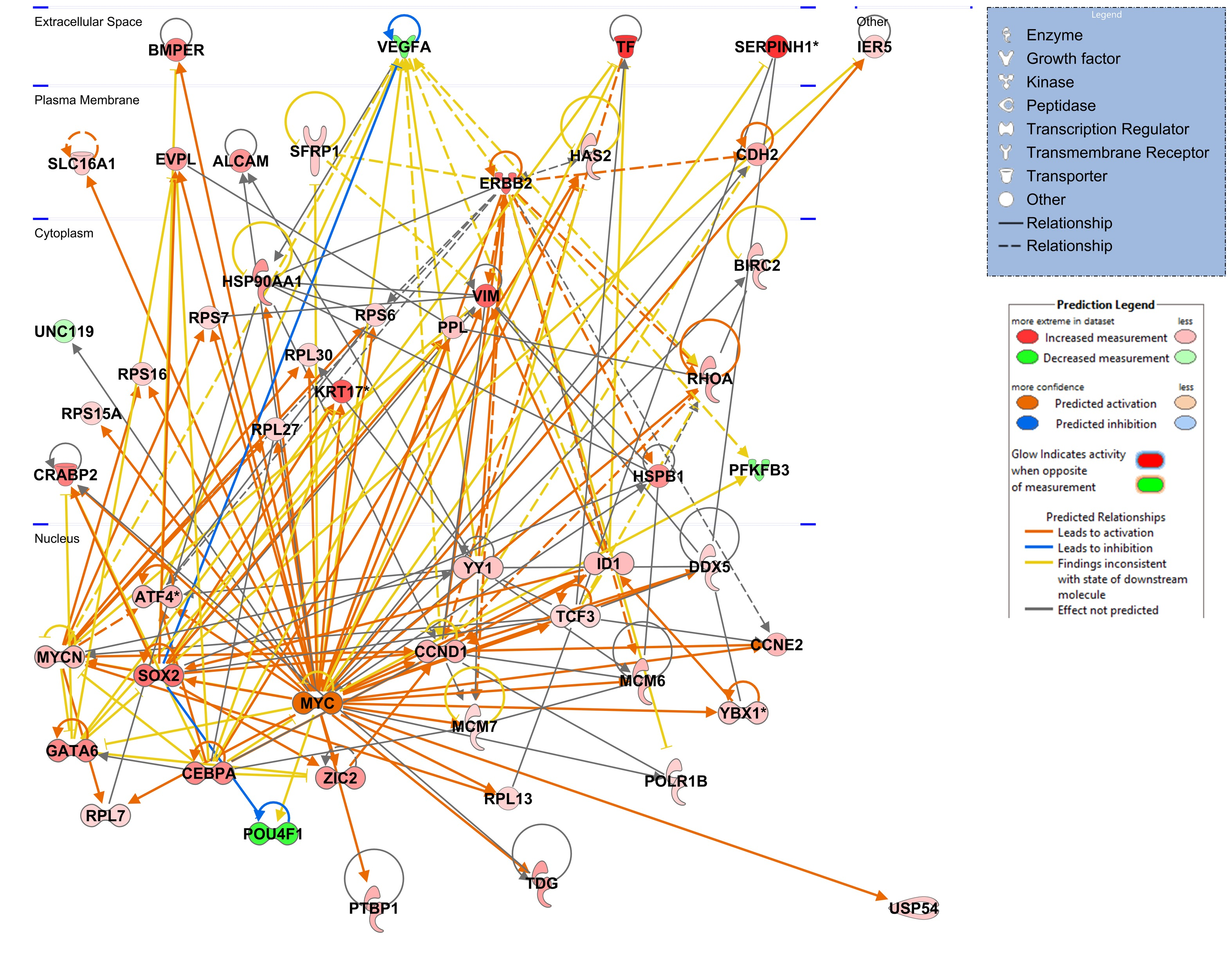

Supplement: S5 Fig — Myc target gene networks and interactions, presented in a subcellular layout as part of the 10.9 mGy/h group. (TIF) [file pone.0179259.s010.tif]

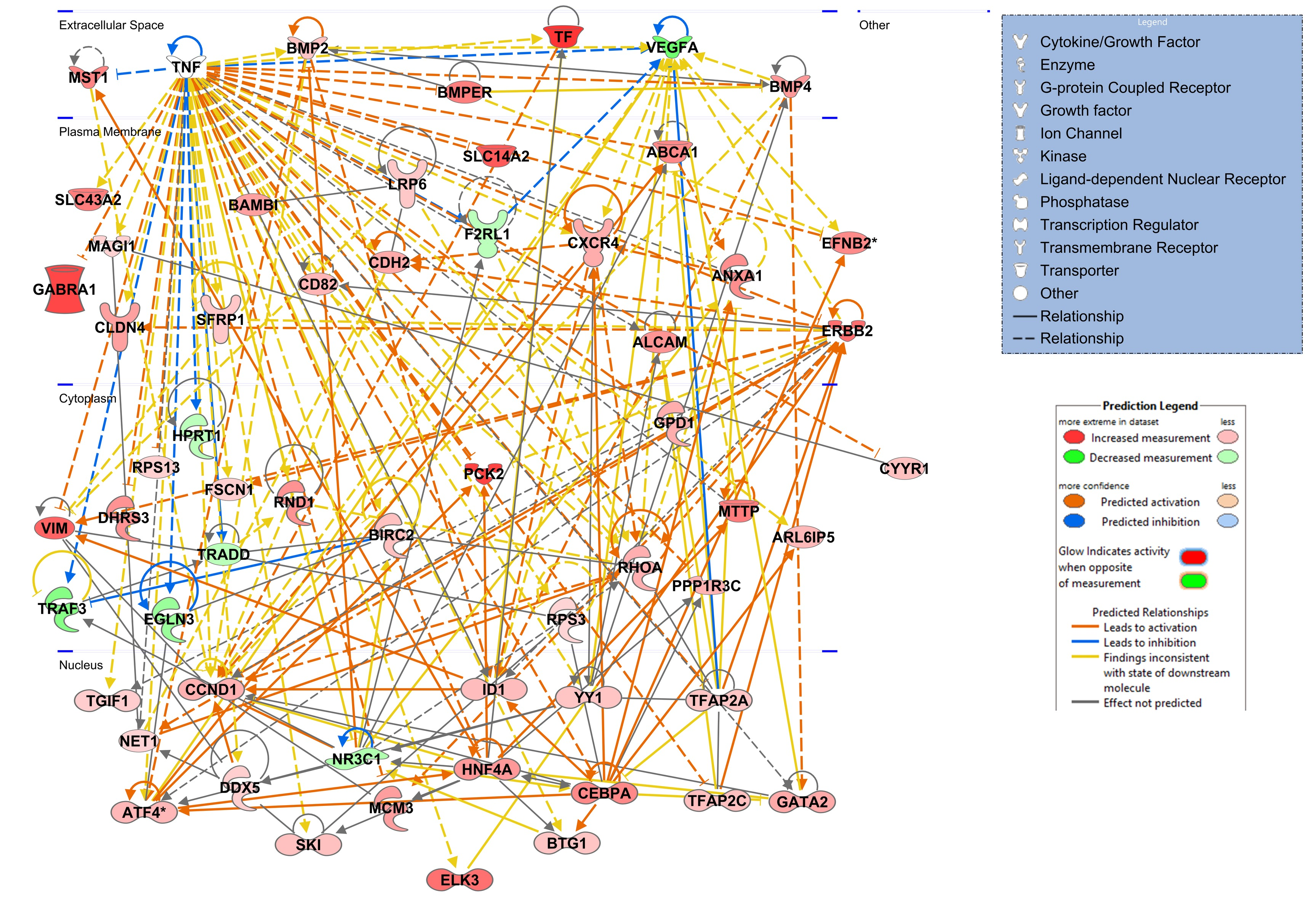

Supplement: S6 Fig — TNF target gene networks and interactions, presented in a subcellular layout as part of the 10.9 mGy/h group. (TIF) [file pone.0179259.s011.tif]

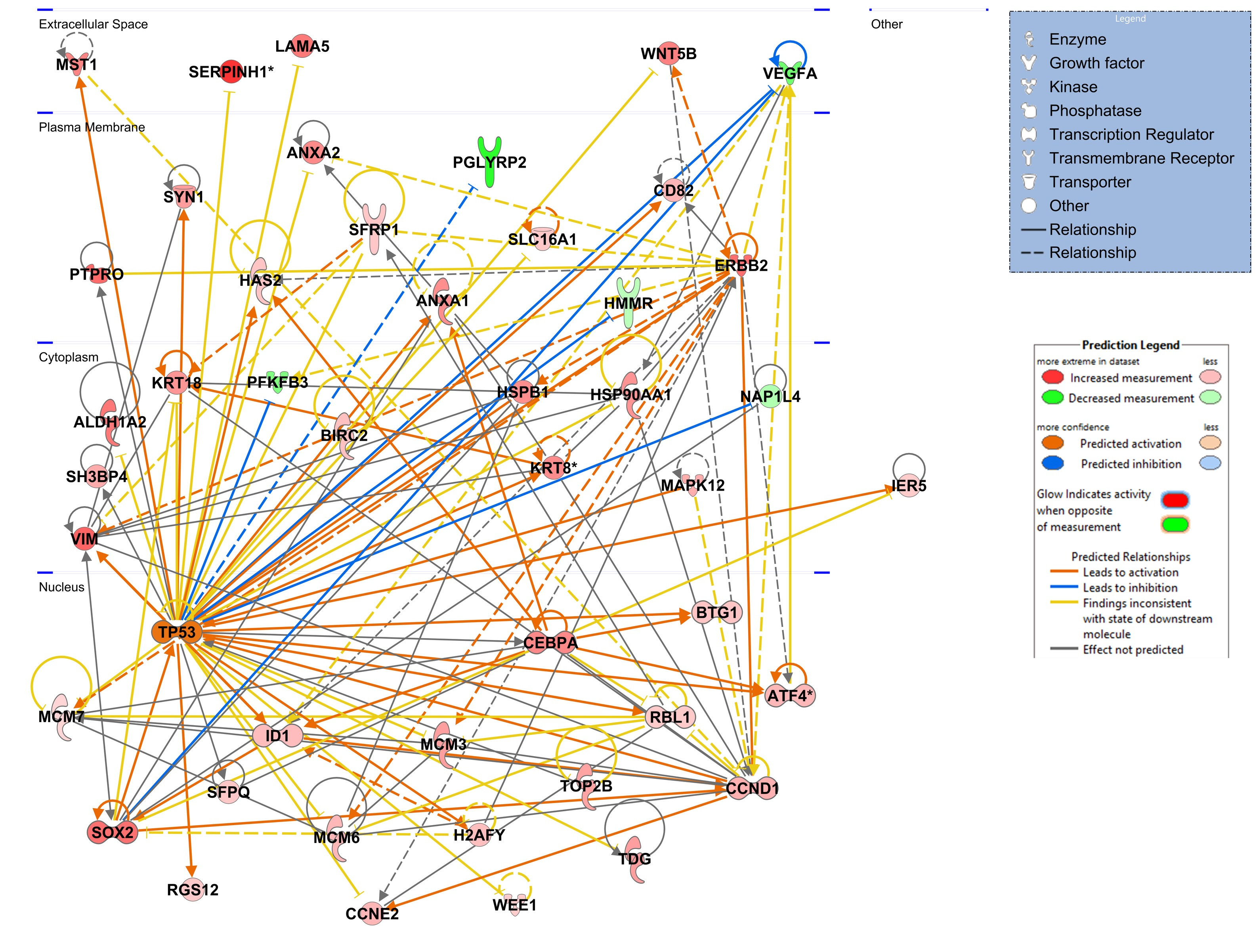

Supplement: S7 Fig — Tp53 target gene networks and interactions, presented in a subcellular layout as part of the 10.9 mGy/h group. (TIF) [file pone.0179259.s012.tif]

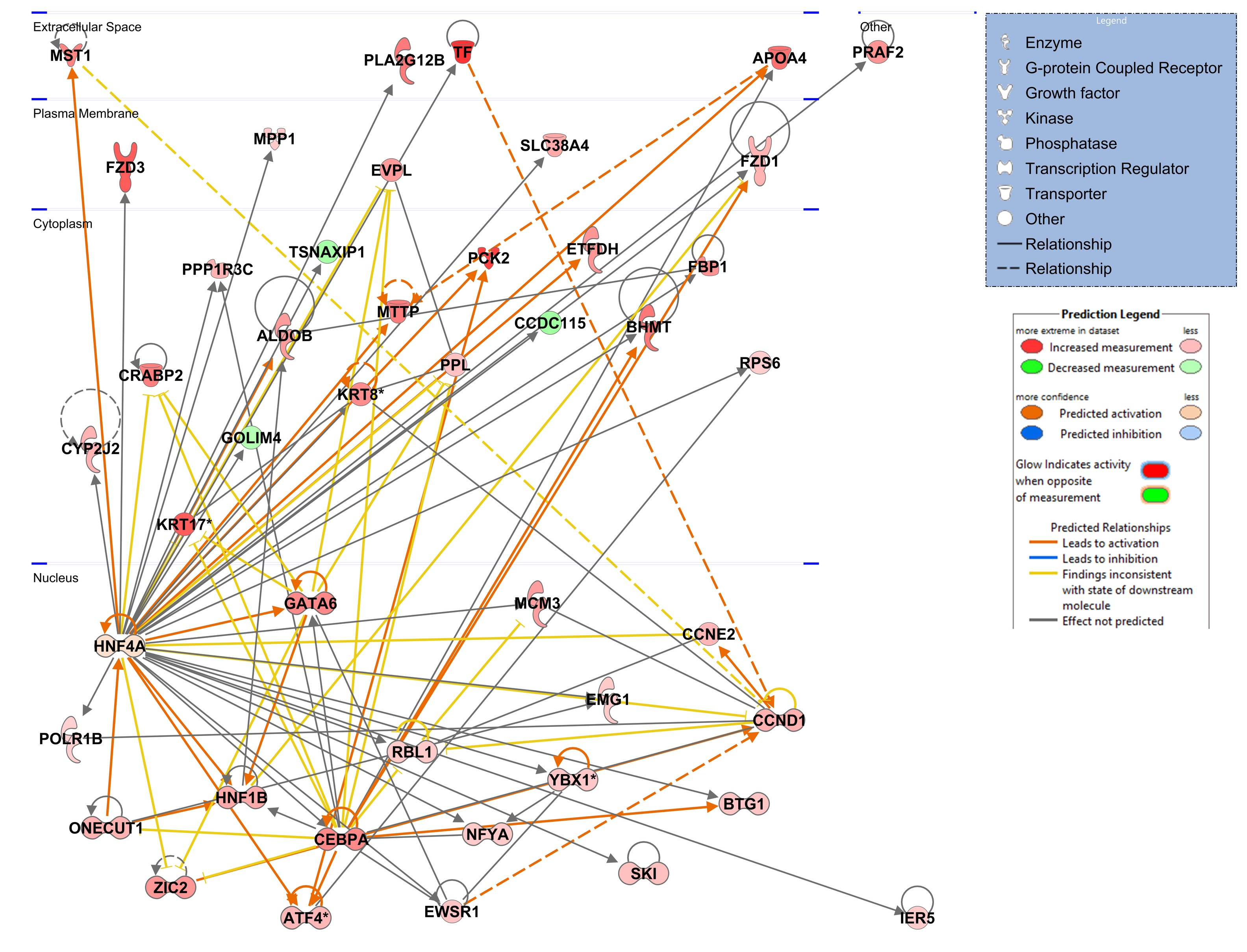

Supplement: S8 Fig — Hnf4a target gene networks and interactions, presented in a subcellular layout as part of the 10.9 mGy/h group. (TIF) [file pone.0179259.s013.tif]

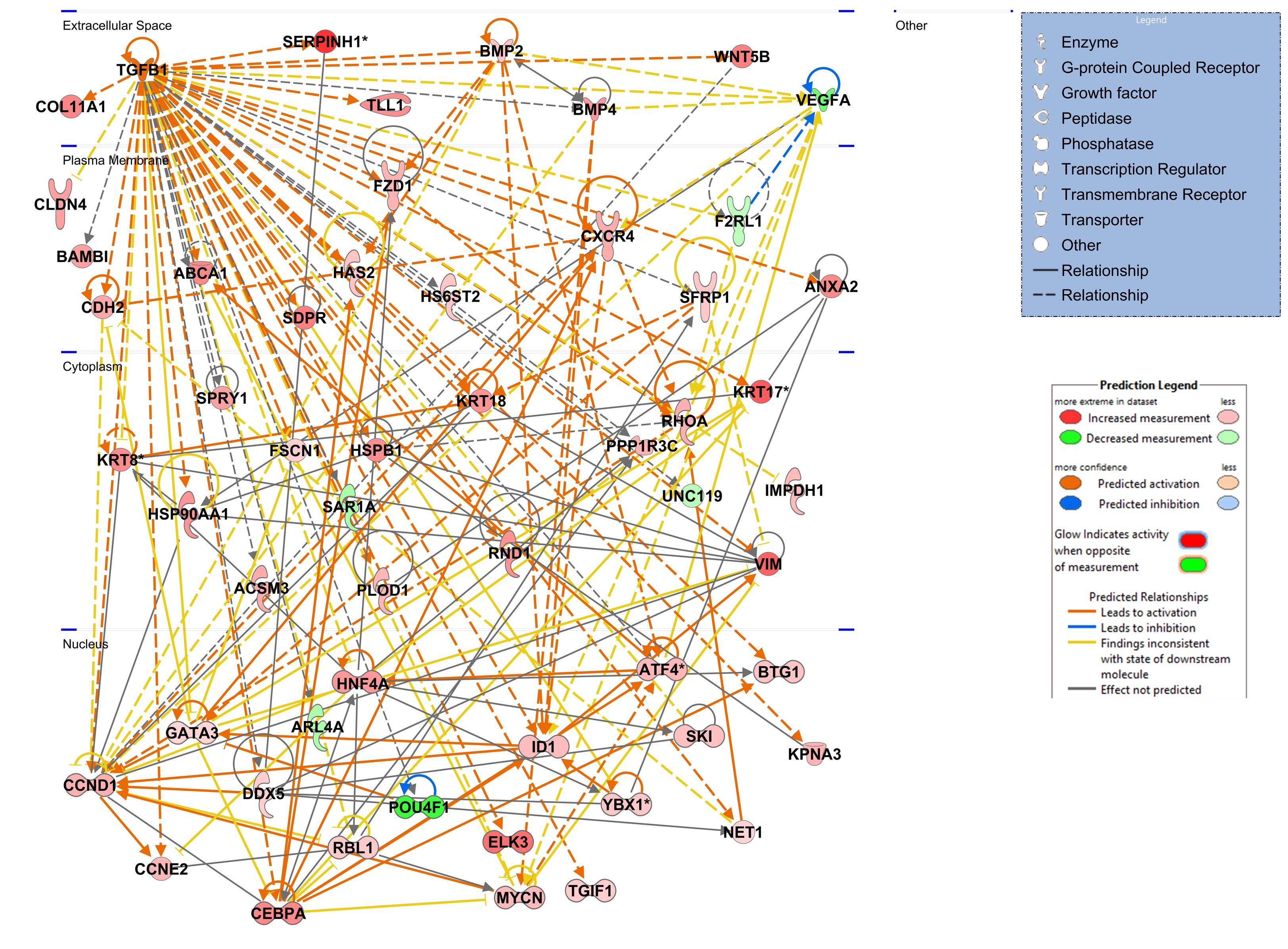

Supplement: S9 Fig — TGFB1 target gene networks and interactions, presented in a subcellular layout as part of the 10.9 mGy/h group. (TIF) [file pone.0179259.s014.tif]

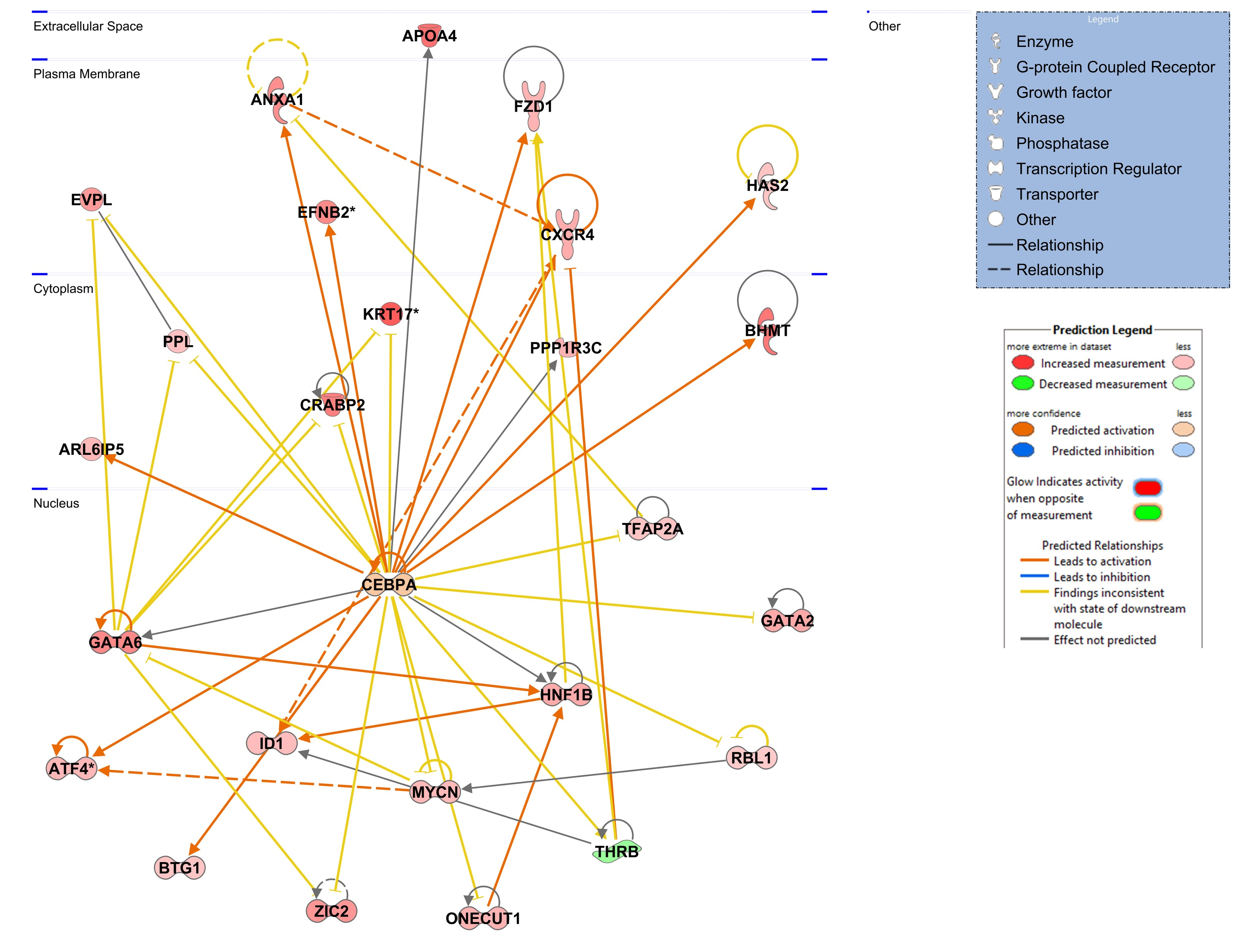

Supplement: S10 Fig — Cebpa target gene networks and interactions, presented in a subcellular layout as part of the 10.9 mGy/h group. (TIF) [file pone.0179259.s015.tif]

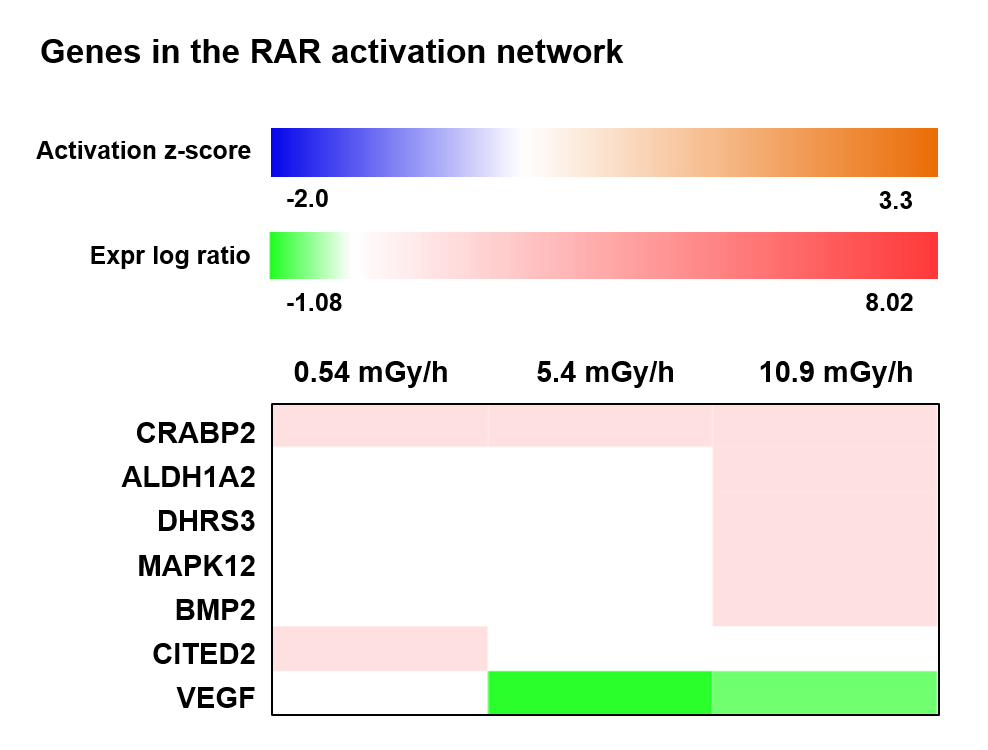

Supplement: S11 Fig — (TIF) [file pone.0179259.s016.tif]

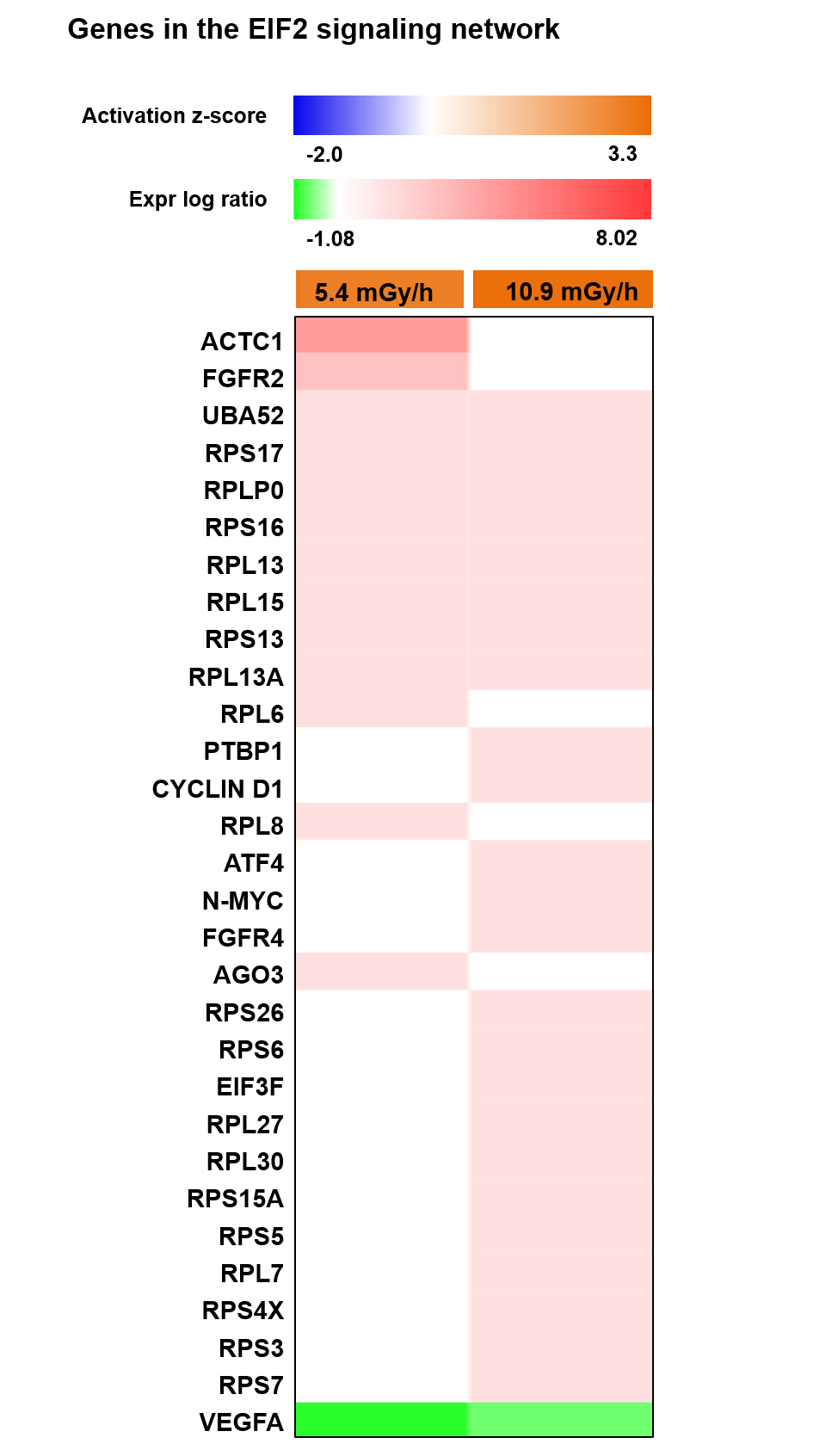

Supplement: S12 Fig — (TIF) [file pone.0179259.s017.tif]

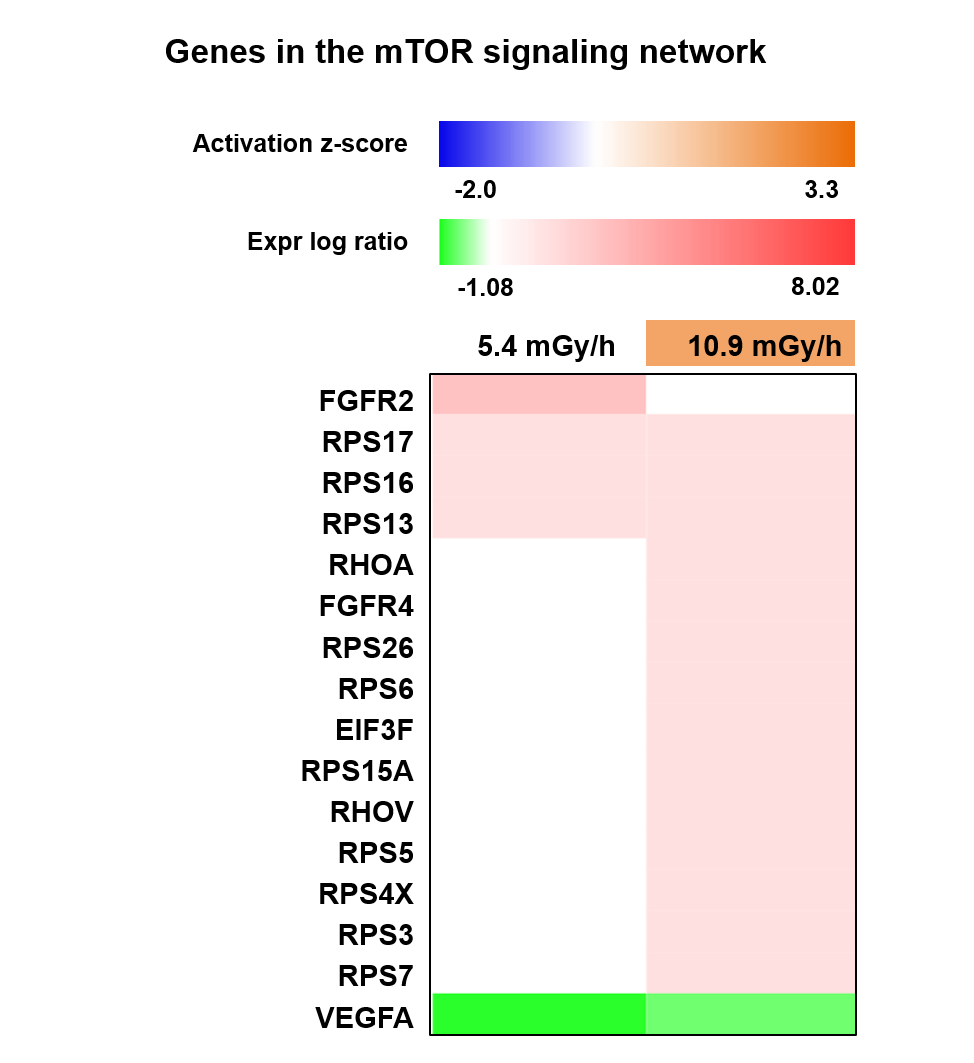

Supplement: S13 Fig — (TIF) [file pone.0179259.s018.tif]

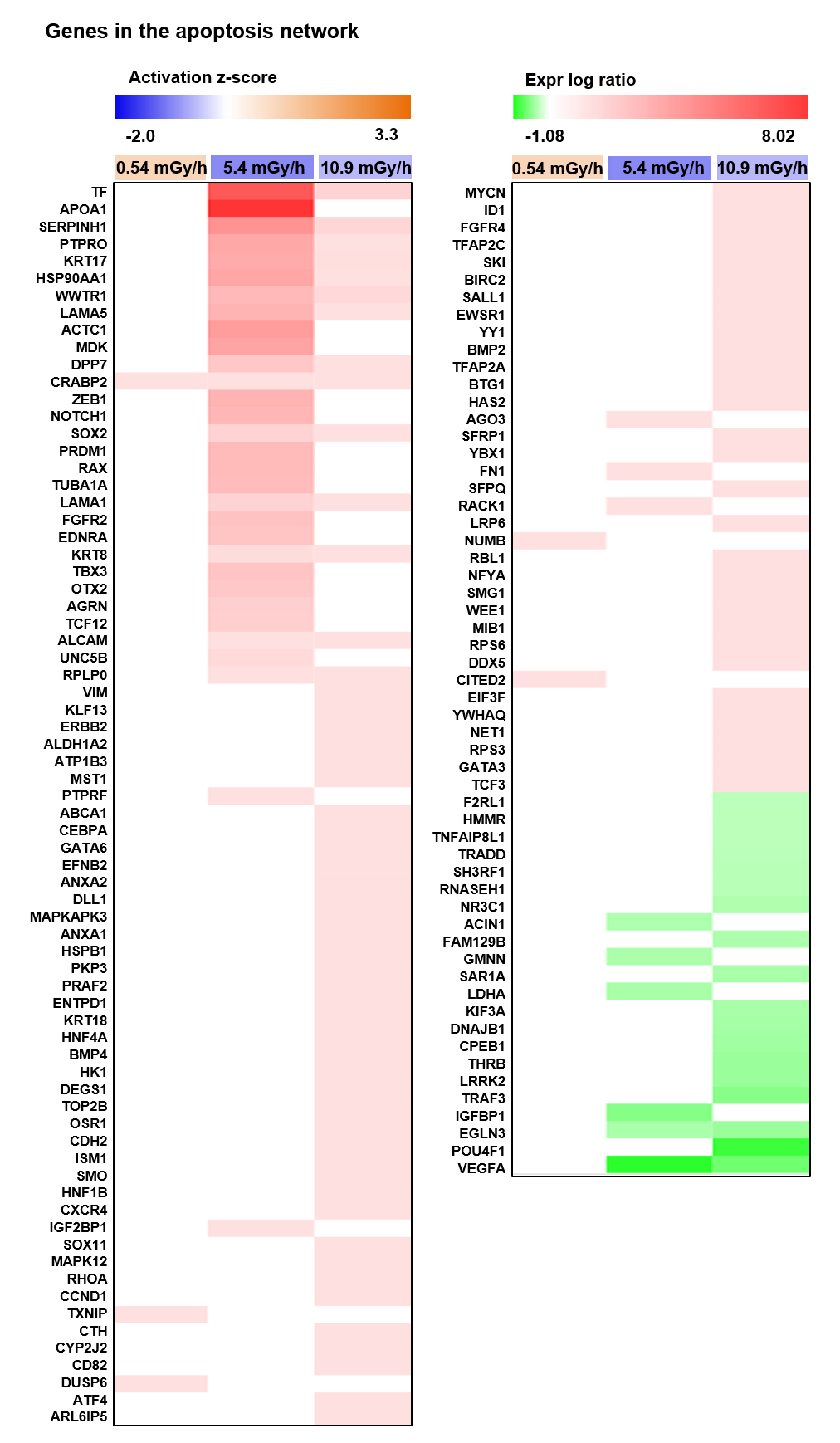

Supplement: S14 Fig — (TIF) [file pone.0179259.s019.tif]

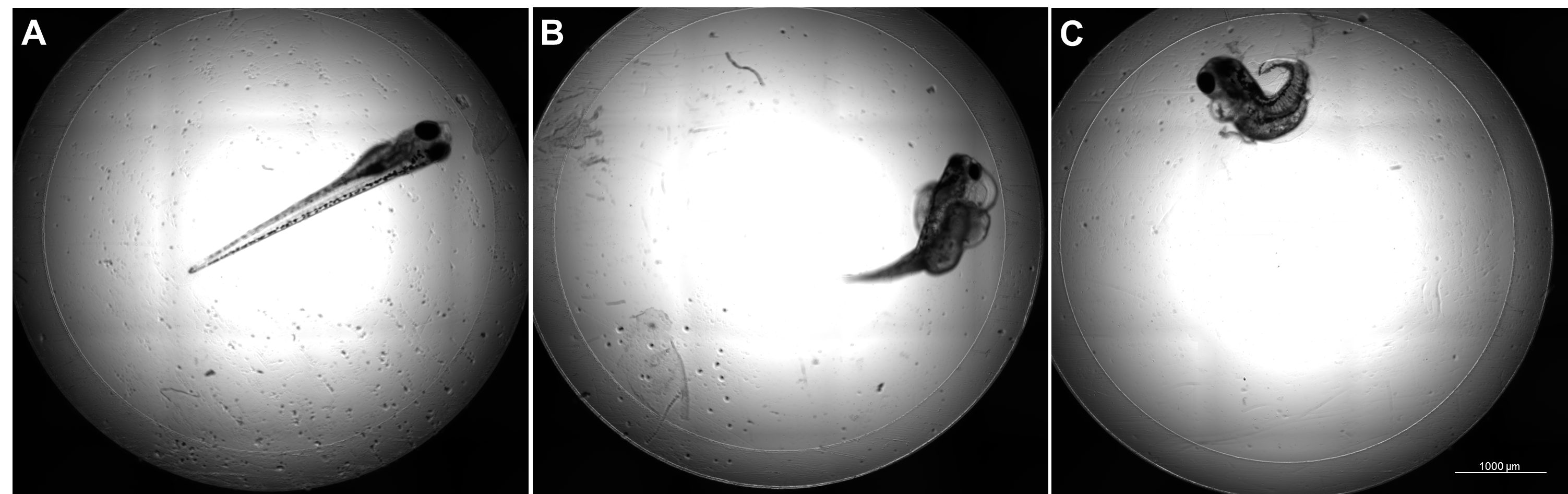

Supplement: S15 Fig — The observations were done at 96 hours post fertilization (hpf). A. Control larva showing normal development; B-C. Larvae exposed to 38 mGy/h for 92 hours (Group “B”), demonstrating general developmental defects and short-tails. (TIF) [file pone.0179259.s020.tif]
